# Overcoming the Shield: Mechanisms of Resistance to DNA Repair Inhibitors and Strategies to Restore Synthetic Lethality

**DOI:** 10.32604/or.2026.081879

**Published:** 2026-10-28

**Authors:** Kannan Sridharan, Ondrej Fiala, Gowri Sivaramakrishnan, Mimma Rizzo, Matteo Santoni

**Affiliations:** 1 Department of Pharmacology and Therapeutics, College of Medicine and Health Sciences, Arabian Gulf University, Manama, Bahrain; 2 Department of Oncology and Radiotherapeutics, Faculty of Medicine and University Hospital in Pilsen, Charles University, Pilsen, Czech Republic; 3 Biomedical Center, Faculty of Medicine in Pilsen, Charles University, Pilsen, Czech Republic; 4 ARON Research Foundation ETS, Galleria del Commercio n. 6, Macerata, Italy; 5 Bahrain Defence Force Royal Medical Services, Riffa, Bahrain; 6 Medical Oncology Unit, Azienda Ospedaliera Universitaria Consorziale Policlinico di Bari, Bari, Italy; 7 Medical Oncology Unit, Macerata Hospital, Macerata, Italy

**Keywords:** BRCA1/BRCA2 mutations, drug resistance, poly (ADP-ribose) polymerase (PARP) inhibitors, prostate cancer, synthetic lethality

## Abstract

The emergence of resistance to poly (ADP-ribose) polymerase (PARP) inhibitors poses a significant obstacle in treating cancers characterized by BReast CAncer gene (*BRCA*) mutations or homologous recombination deficiency. Despite the substantial clinical benefits brought by drugs such as olaparib, niraparib, and talazoparib, a substantial proportion of tumors ultimately develop resistance. The underlying mechanisms are diverse and include the reconstitution of homologous recombination via secondary BRCA reversion mutations, stabilization of replication forks, enhanced drug efflux mediated by p-glycoproteins, and structural or functional alterations in PARP1 itself. A thorough grasp of these resistance pathways is critical for the design of effective therapeutic interventions. Emerging strategies aimed at countering resistance involve combinatorial regimens incorporating inhibitors of Ataxia telangiectasia and Rad3-related (ATR), WEE1, checkpoint kinase 1 (CHK1), or immune checkpoint blockade, and other agents that target DNA damage response (DDR) networks. Furthermore, next-generation PARP inhibitors and optimized drug scheduling paradigms are under investigation to reestablish tumor sensitivity. Refined, biomarker-guided patient selection and advanced molecular surveillance may further optimize treatment outcomes and postpone the onset of resistance. This review offers a mechanism-centric synthesis of resistance to DNA repair inhibitors and the therapeutic strategies being developed to circumvent it.

## 1 Introduction

The development of DNA repair inhibitors marks a major advance in precision oncology by exploiting synthetic lethality, targeting tumor-specific vulnerabilities within the DNA damage response (DDR) network [1]. Poly (ADP-ribose) polymerase (PARP) inhibitors exemplify this paradigm, selectively targeting tumors with homologous recombination deficiency (HRD), particularly those with BReast CAncer genes 1 or 2 (BRCA1/2) mutations [2]. In pivotal trials, PARP inhibitors significantly extended progression-free survival [3]; for example, in ovarian cancer, median progression-free survival improved from 5–6 months to 16–21 months [4,5,6]. Efficacy has since expanded to breast, pancreatic, and prostate cancers, including tumors with alterations in other homologous recombination repair (HRR) genes [7,8]. However, 40–70% of patients develop intrinsic or acquired resistance, limiting long-term efficacy [9]. Similar patterns are emerging for Ataxia telangiectasia and Rad3-related (ATR), Ataxia-Telangiectasia Mutated (ATM), and Checkpoint kinase 1 (CHK1) inhibitors [10]. This review provides a mechanism-oriented synthesis of resistance to DNA repair inhibitors and strategies to overcome it, integrating clinical and molecular insights to inform biomarker-driven, evolution-aware treatments.

## 2 DNA Repair Inhibitors and Their Mechanisms of Action

DNA repair inhibitors target key nodes in the DDR network, including PARP, ATM, ATR, CHK1, WEE1, and DNA-dependent protein kinase catalytic subunit (DNA-PKcs). PARP1 binds single-strand breaks and performs PARylation to recruit repair effectors; PARP inhibitors exploit synthetic lethality with HRD in BRCA1/2-mutant tumors [11]. ATM, DNA-PKcs, and ATR orchestrate checkpoint control; ATR inhibitors (ceralasertib, berzosertib) show clinical promise [12]. CHK1 inhibition abrogates checkpoints in p53-defective tumors [13], while DNA-PKcs inhibition forces error-prone repair [14]. Emerging targets include polymerase theta (POLQ), a synthetic lethal target in HRD tumors to overcome PARP inhibitor resistance [15], and WEE1, whose inhibition induces mitotic catastrophe [16].

## 3 Mechanisms of Resistance to DNA Repair Inhibitors

Resistance to DNA repair inhibitors, particularly PARP inhibitors, can be intrinsic or acquired. Mechanisms are diverse, spanning genetic, epigenetic, and pathway-level adaptations.

### 3.1 Restoration of HR

This is the most prevalent resistance mechanism, directly countering the principle of synthetic lethality in HRD tumors [17].

- **BRCA1/2 Reversion Mutations:** Therapeutic pressure frequently selects for secondary mutations that restore the BRCA1/2 open reading frame, yielding a functional protein (Fig. 1) [18]. A systematic review identified exon 11 as a common site for these events [19]. Detecting these reversions in circulating tumor DNA (ctDNA) offers a non-invasive strategy for real-time resistance monitoring, often preceding radiographic progression [20].

**Figure 1:**
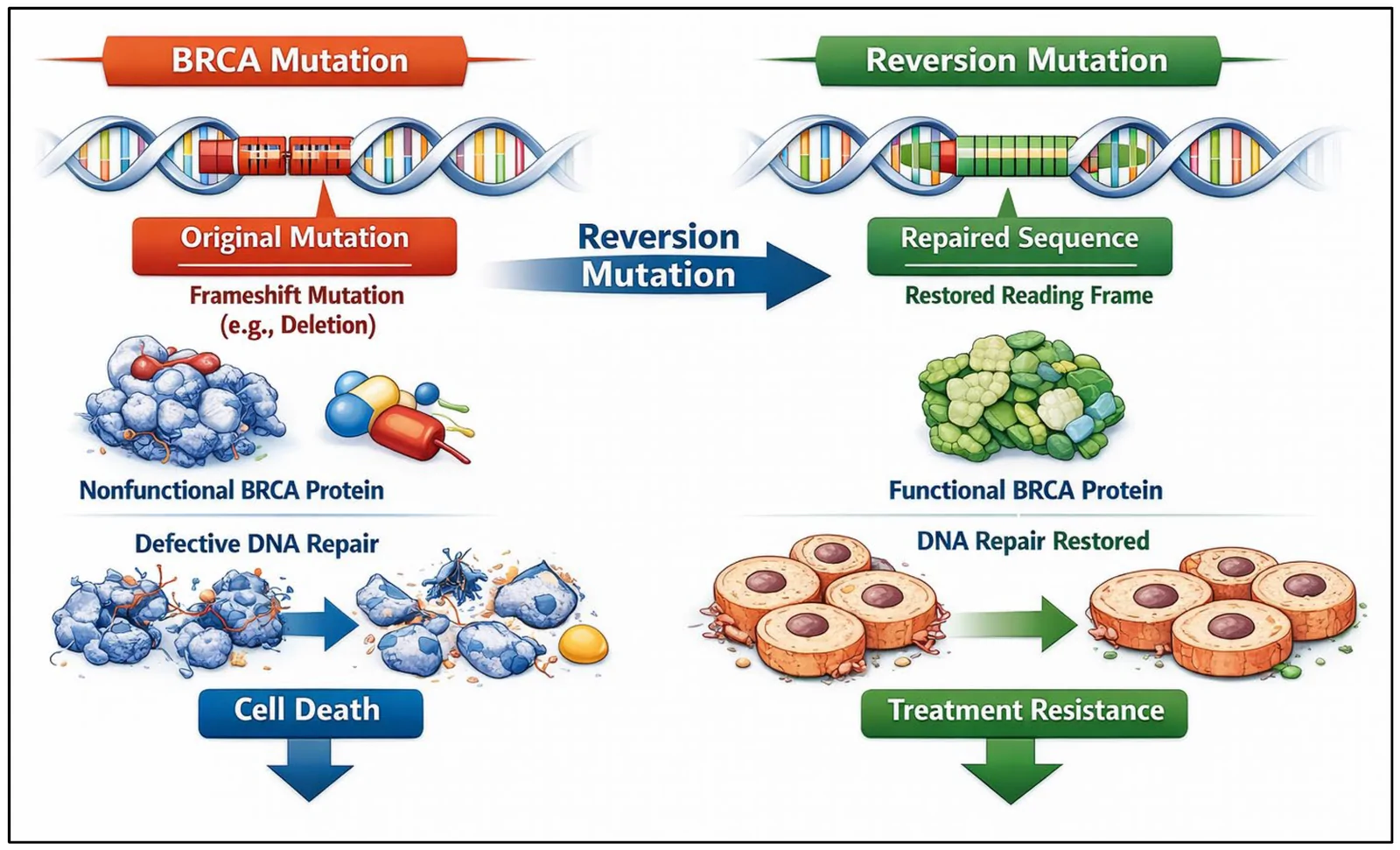
Biological rationale and clinical relevance of *BRCA* reversion mutations.

Schematic illustration of BRCA1/2 reversion-mediated resistance: an initial pathogenic BRCA1/2 alteration causes homologous recombination deficiency and PARP inhibitor sensitivity, followed by acquisition of a reversion event that restores the BRCA open reading frame/function, reconstitutes HR repair, and results in PARP inhibitor resistance.

- **Epigenetic Reversal:** In tumors where *BRCA1* silencing is due to promoter hypermethylation, resistance can arise from demethylation and subsequent protein re-expression [21]. Unlike genetic reversions, epigenetic changes are potentially reversible, though therapeutic modulation remains challenging [22].
- **Loss of p53-binding protein 1/Shieldin Function:** p53-binding protein 1 (53BP1) and its downstream effectors (shieldin complex) restrict DNA end resection, channeling repair toward NHEJ and enforcing HRD in *BRCA1*-deficient cells [23]. Loss of any component restores HR and confers PARP inhibitor resistance, making this axis a therapeutic target for re-sensitization.

### 3.2 Replication Fork Stabilization

In *BRCA*-deficient cells, stalled replication forks are degraded, contributing to lethality. Resistance can arise from stabilizing these forks by preventing their degradation, independent of HR restoration [24] or loss of epigenetic regulators that promote fork stability and conferring resistance [25].

### 3.3 Other Mechanisms

- **Drug Efflux:** Upregulation of ATP-binding cassette (ABC) transporters, such as P-glycoprotein, can reduce intracellular drug concentration [26].
- **PARP1 Alterations:** Mutations altering PARP1’s DNA trapping capability can confer resistance [27].
- **Upregulation of Alternative Repair:** Increased expression of POLQ, a key effector of Microhomology-Mediated End-Joining (MMEJ), provides a backup repair pathway in HR-deficient cells, reducing PARP inhibitor sensitivity [28]. This underpins the rationale for developing POLQ inhibitors.
- **Ferroptosis induction:** Finally, emerging evidence implicates ferroptosis, an iron-dependent, lipid peroxidation-driven form of regulated cell death, in the modulation of resistance to DNA-damaging agents. Pharmacological induction of ferroptosis can overcome acquired chemotherapy resistance across multiple cancer types, and growing data suggest that ferroptosis pathways intersect with DDR inhibitor responses [29]. Although direct evidence for ferroptosis induction reversing DDR inhibitor resistance remains preliminary, this represents an intriguing future direction that expands the synthetic lethality framework beyond apoptosis-dependent mechanisms.

### 3.4 Resistance Mechanisms Specific to ATR Inhibitors

Resistance to ATR inhibitors arises through convergent mechanisms: restoration of downstream DNA damage response signaling, activating mutations or amplification of replication fork stabilization genes, upregulation of alternative replication stress pathways, and loss of nonsense-mediated decay factors [30,31]. Whether these mutations emerge in patients remains under investigation, as longitudinal biopsy and ctDNA studies from ATR inhibitor trials are still maturing.

### 3.5 Resistance Mechanisms Specific to CHK1 Inhibitors

Regarding resistance to CHK1 inhibitors, prolongation of G2 delay induced by lower CDK1/CyclinB1 activity, overexpression or amplification of the related kinase checkpoint kinase 2 (CHK2), which can partially compensate for CHK1 loss in checkpoint control, loss of the phosphatase (Cell Division Cycle 25A) or mutations in cell cycle regulators such as cyclin E or CDK2, and upregulation of efflux transporters, particularly P-glycoprotein, were the detected mechanisms [32].

### 3.6 Resistance Mechanisms Specific to WEE1 Inhibitors

Regarding the resistance mechanisms for WEE1 inhibitors, upregulation of the related kinase Protein Kinase, Membrane-Associated Tyrosine/Threonine 1 (PKMYT1) also known as Myt1 and mammalian target of rapamycin were identified mechanisms [33].

### 3.7 Resistance Mechanisms Specific to DNA-PKcs Inhibitors

DNA-PKcs resistance involves upregulation of alternative end joining pathways, particularly POLQ-mediated MMEJ and loss of the Ku70/Ku80 heterodimer were identified mechanisms [34].

## 4 Resistance beyond the Cancer Cell: Microenvironmental Modulation of Synthetic Lethality

The tumor microenvironment (TME) actively reinforces tumor-intrinsic resistance pathways, creating an adaptive niche that mitigates the therapeutic vulnerability created by DNA repair inhibition. Hypoxia, metabolic plasticity, and immune remodeling represent critical extrinsic determinants of therapeutic outcome.

### 4.1 Hypoxia and Repair Pathway Plasticity

Hypoxia regulates DNA repair via hypoxia-inducible factors (HIF1α and HIF2α). Acute hypoxia suppresses HR by repressing BRCA1 and Radiation Deficient 51 (RAD51), inducing a context-dependent ‘BRCAness’ phenotype that increases PARP inhibitor sensitivity [35,36,37]. Conversely, chronic hypoxia promotes adaptive DDR (DNA damage response) rewiring, alters repair pathway use, and modulates replication fork stability, reducing reliance on S-phase-coupled repair processes [38]. Hypoxia-driven reductions in proliferation limit S-phase cells, attenuating DDR inhibitor efficacy [37]. Additionally, hypoxic niches harbor drug-tolerant persister cells that survive initial PARP inhibitor exposure, linking microenvironmental stress to non-genetic resistance and shaping synthetic lethality [39].

### 4.2 Metabolic Rewiring and NAD^+^ Homeostasis

PARP1 consumes NAD^+^ during PARylation; sustained activation depletes NAD^+^, compromising ATP production and engaging AMP-activated protein kinase (AMPK) signaling [40,41]. Cancer cells counter this by enhancing nicotinamide phosphoribosyltransferase (NAMPT)-dependent NAD^+^ salvage, preserving PARylation capacity and driving PARP inhibitor resistance [42,43]. Dual NAMPT and PARP inhibition induces NAD^+^ depletion and amplifies DNA damage preclinically [42,44]. Beyond NAD^+^ metabolism, glutamine metabolism supports nucleotide biosynthesis and redox homeostasis via the tricarboxylic acid cycle and glutathione production, facilitating DNA repair [45,46]. The pentose phosphate pathway (PPP) generates ribose-5-phosphate for nucleotide synthesis and NADPH for antioxidant defenses, limiting oxidative DNA damage [47]. Lipid metabolism also influences genotoxic stress responses via fatty acid oxidation and chromatin regulation [48]. These interconnected circuits modulate replication stress and repair pathway choice, though their context-dependent contributions to PARP inhibitor resistance remain an active area of investigation.

### 4.3 Immune Remodeling: Checkpoint Induction and T-Cell Dysfunction

DDR inhibition generates cytosolic DNA, activating cyclic GMP-AMP synthase (cGAS) and stimulator of interferon genes (STING), leading to type I interferon production and enhanced tumor immunogenicity [49,50]. PARP inhibitors increase C-X-C motif chemokine ligand 10 (CXCL10) and C-C motif chemokine ligand 5 (CCL5), promoting CD8^+^ T-cell infiltration. However, sustained DDR signaling also upregulates programmed death-ligand 1 (PD-L1) via ATM-ATR-CHK1 signaling, though PD-L1 assessment is confounded by spatial heterogeneity and transient expression [49,50]. Persistent inflammation drives T-cell exhaustion and recruits immunosuppressive myeloid cells. PARP inhibitors also skew tumor-associated macrophages (TAMs) toward an M2-like immunosuppressive phenotype via metabolic remodeling (oxidative and fatty acid oxidation), counterbalancing cGAS-STING effects [51]. Early-phase trials combining PARP inhibitors with PD-1/PD-L1 inhibitors show heterogeneous responses, reflecting this duality. Optimal integration requires enhanced biomarker strategies, spatial-temporal profiling, and optimized scheduling to sustain antitumor immunity [51].

### 4.4 TME Reprogramming

The dynamic reprogramming of the TME in response to DNA damage signaling and therapeutic pressure represents an emerging layer of resistance that intersects with both DDR inhibitor efficacy and immune checkpoint response [52]. Beyond the static characterization of immune cell infiltrates discussed above, this framework emphasizes that cancer-associated fibroblasts (CAFs), endothelial cells, and myeloid populations undergo functional polarization in response to PARP inhibitor-induced genotoxic stress. Specifically, PARP inhibition can drive CAFs toward a senescence-associated secretory phenotype (SASP), characterized by the release of interleukin 6 (IL-6), IL-8, and Transforming growth factor-beta (TGFβ), which paradoxically supports residual tumor cell survival through paracrine activation of Nuclear factor kappa-light-chain-enhancer of activated B cells (NF-κB) and Signal Transducer and Activator of Transcription 3 (STAT3) signaling [53]. Simultaneously, endothelial cell reprogramming alters vascular permeability and adhesion molecule expression, influencing both drug delivery and immune cell extravasation. The therapeutic implication is that targeting TME components, for example, using Focal Adhesion Kinase (FAK) inhibitors to reverse CAF-mediated resistance, or disrupting C-X-C motif chemokine receptor 2 (CXCR2) signaling to block myeloid-derived suppressor cell recruitment [54], may enhance the efficacy of DDR inhibitors in otherwise resistant tumors. This TME-centric perspective expands the synthetic lethality concept beyond tumor-cell-autonomous mechanisms, suggesting that combination strategies must account for the bidirectional crosstalk between cancer cells and their stromal niche.

## 5 Rewiring Vulnerabilities: Novel Synthetic Lethal Strategies to Bypass Resistance

Acquired resistance to DNA repair inhibitors is indicative of adaptive rewiring, rather than full restoration of the original pre-treatment state. Despite the restoration of HR, the stabilization of replication forks, or the restoration of metabolic balance, resistant tumors frequently persist by maintaining elevated basal replication stress and enhanced reliance on cell-cycle checkpoints. The identification of these persistent liabilities has given rise to novel therapeutic opportunities. Consequently, contemporary strategies are pivoted from endeavors to reproduce the original synthetic lethal interaction towards the exploitation of shared vulnerabilities that emerge in resistant tumor states.

### 5.1 Polymerase Theta (POLQ) Targeting in HR-Restored Tumors

POLQ-mediated end joining (TMEJ) remains a critical backup pathway when HR is impaired and often persists as a dependency even after apparent HR restoration, because repair is often incomplete or error-prone and resection/fork dynamics remain abnormal [55]. Small-molecule POLQ inhibitors have demonstrated the capacity to target PARP inhibitor resistance, including resistance driven by 53BP1-shieldin loss. Early-phase clinical development of POLQ inhibitors is ongoing, with first-in-class agents such as ART4215 currently being evaluated in a phase 1/2 trial in advanced solid tumors (NCT04991480), and novobiocin undergoing phase I investigation in patients with HR- or DDR-altered tumors (NCT05687110). Available data from these studies indicate preliminary tolerability and emerging signals of activity in molecularly selected populations, although clinical efficacy remains to be established [56].

The mechanistic rationale is compelling: HR-deficient tumors become addicted to POLQ for double-strand break repair, and even when HR is partially restored through reversion mutations or suppressor mutations, this addiction may persist due to residual genomic instability. POLQ inhibitors therefore represent a strategy to convert the historical fact of HR deficiency into a persistent therapeutic vulnerability, independent of current HR functional status.

### 5.2 ATR and CHK1 Inhibition in Checkpoint-Dependent Resistance

PARP inhibitor-resistant tumors frequently exhibit elevated markers of replication stress, including accumulation of replication protein A (RPA) and sustained activation of ATR [57]. Even when HR is partially restored, the architecture of replication forks and the dynamics of origin firing often remain aberrant, which increases reliance on CHK1 signaling to prevent fork collapse and coordinate S-phase progression [58].

Early-phase clinical trials have demonstrated activity of ATR inhibitors such as ceralasertib (AZD6738) and elimusertib (BAY1895344) in PARP inhibitor-pretreated populations [59,60]. Preliminary *in vivo* investigations indicate that ATR inhibition in PARP inhibitor-resistant models triggers replication catastrophe, characterized by extensive fork collapse, double-strand break accumulation, and mitotic aberrations [61]. By targeting persistent checkpoint dependence rather than homologous recombination itself, ATR-CHK1 inhibition exploits a vulnerability that is shared by heterogeneous resistance mechanisms, offering a potential “one-size-fits-most” approach for PARP inhibitor-resistant disease.

### 5.3 WEE1 Inhibition and Replication Stress Catastrophe

WEE1 kinase restrains the activity of cyclin-dependent kinase 1 and 2 (CDK1/2), thereby enforcing intra-S and G2/M checkpoints in response to DNA damage and replication stress. Inhibition of WEE1 abrogates these safeguards, driving CDK-dependent unscheduled origin firing, rapid depletion of nucleotide pools, and RPA exhaustion, which culminate in replication catastrophe and premature mitotic entry [62,63].

Tumors that have acquired resistance to DDR inhibitors frequently exhibit elevated basal replication stress, thus rendering them particularly dependent on checkpoint control for survival. The WEE1 inhibitor adavosertib exhibits clinical activity in selected genomically unstable and replication stress-high contexts, and combination strategies with PARP inhibitors or ATR inhibitors are being actively evaluated [64,65]. From a mechanistic perspective, WEE1 inhibition converts compensated replication stress tolerance into lethal genome fragmentation, a process particularly evident in tumors already burdened by DDR inhibitor-induced instability [63].

### 5.4 Epigenetic Re-Sensitization through Chromatin Modulation

Adaptive resistance to DDR inhibitors frequently involves epigenetic reprogramming, which reshapes chromatin accessibility at loci controlling DNA repair and replication stress responses. Transcription is subject to regulation by chromatin-modifying enzymes, including the histone methylase enhancer of zeste homolog 2 (EZH2) and histone deacetylases (HDACs). These enzymes influence the activity of HR factors, replication proteins, and checkpoint regulators, thereby impacting DNA repair pathway choice and replication fork stability. Changes in chromatin compaction and histone modification may, therefore, facilitate partial restoration of HR activity or promotion of replication fork protection, without the necessity for stable genetic alterations [65].

Recent findings suggest that targeting these chromatin regulators has the potential to reverse adaptive resistance states. In preclinical models, EZH2 loss or inhibition alters replication fork protection pathways and contributes to re-sensitization of PARP inhibitor-resistant tumors by disrupting repair factor recruitment and fork stability [66]. Furthermore, HDAC inhibitors have been observed to suppress HR gene expression (including BRCA1 and RAD51), thereby inducing a “BRCAness”-like state. Consequently, these inhibitors restore sensitivity to PARP inhibition and enhance the efficacy of PARP inhibitor combinations [67,68]. These findings indicate that chromatin-targeted therapies might facilitate the reopening of synthetic lethal vulnerabilities by reversing epigenetic programs underlying resistance [69].

## 6 Biomarkers for Patient Stratification and Resistance Monitoring

Resistance to DNA repair inhibitors is dynamic. Tumors can rewire repair processes, expand resistant subclones, and alter drug sensitivity over time and across metastatic sites. Therefore, a complementary approach integrating longitudinal monitoring is essential for effective patient stratification and resistance management [70,71]. Functional assessment of HR status can capture current repair capacity [72,73], while historical genomic footprints of repair deficiency (genomic scars and mutational signatures) provide evidence of prior HRD states [74,75]. Finally, spatial and dynamic context can be supported by imaging approaches, including PARP-target engagement and PARP inhibitor-derived probes [76,77].

### 6.1 Circulating Tumor DNA (ctDNA) for Detecting BRCA Reversion Mutations

#### 6.1.1 Rationale

BRCA1/2 reversion mutations are a canonical mechanism of acquired resistance to PARP inhibitors and platinum agents. These secondary events restore the open reading frame, often via indels or complex rearrangements, and can partially or fully reconstitute HR, thereby undermining synthetic lethality [78]. Importantly, reversions frequently arise as multiple parallel subclones, representing an evolutionary solution under PARP inhibitor selection pressure. This polyclonality increases vulnerability to sampling bias with single-site tissue biopsy. Circulating tumor DNA (ctDNA), by contrast, can capture this heterogeneity and enables longitudinal monitoring across treatment and progression [78,79,80].

#### 6.1.2 Clinical Evidence

The strongest clinical evidence comes from treatment of high-grade serous ovarian carcinoma (HGSOC) with PARP inhibitors. Analysis of cell-free DNA (cfDNA) showed that BRCA reversions detected in ctDNA are associated with reduced clinical benefit and can be enriched or newly detected at progression [79]. Earlier research also revealed various BRCA reversions in cfDNA from ovarian and breast cancers resistant to platinum and/or PARP inhibitors [78]. In prostate cancer, polyclonal BRCA2 reversions have been detected in ctDNA, highlighting convergent evolution and the clinical necessity of anticipating HR restoration as an important resistance mechanism [80,81].

#### 6.1.3 Practical Implementation

ctDNA testing for BRCA reversion mutations is clinically useful at three practical time points: (1) at baseline, before initiation of PARP inhibitor therapy, to confirm the original pathogenic BRCA alteration and, rarely, identify pre-existing low-level reversions or mosaic sub clonality that may blunt initial benefit [78,79]; (2) at suspected progression, which typically provides the highest yield for acquired reversion mutation detection and is the most actionable moment for mechanism-guided selection of subsequent systemic therapy [78,79,82]; and (3) optional early on-treatment sampling (4–8 weeks after PARP inhibitor initiation), which can provide information in cases of high tumor shedding to capture early clonal shifts but remains strongly dependent on tumor fraction and assay characteristics [70,71,83]. In this context, ctDNA-based detection of BRCA reversion mutations may also inform enrollment into trials exploring PARP inhibitor rechallenge versus alternative DDR-targeting combination strategies.

#### 6.1.4 Beyond ctDNA: Epigenetic Tracking of Urinary DNA Methylation

Urinary DNA offers a complementary biofluid for monitoring DDR inhibitor resistance, particularly in urothelial, prostate, and bladder cancers, where it captures tumor-derived material with higher fractional abundance than plasma [84,85]. Urinary DNA methylation analysis enables epigenetic tracking of resistance without requiring detection of rare genetic reversion events; methylation changes at promoters of DDR-related genes can indicate HRD status or acquired resistance via demethylation [84,85]. These changes are quantifiable using targeted bisulfite sequencing or methylation-specific PCR, offering dynamic range suitable for longitudinal monitoring [85]. The non-invasive, patient-administered urine collection reduces logistical burden and enables frequent sampling [84]. However, limitations include variable DNA integrity dependent on processing protocols, need to distinguish tumor-specific methylation from benign urothelial contamination, and insufficient tumor DNA yield in patients without genitourinary tract involvement [84,85]. Integrating urinary DNA methylation monitoring with plasma ctDNA analysis may offer the highest sensitivity for resistance detection [84].

### 6.2 RAD51 Foci as a Functional HR Assay

#### 6.2.1 Rationale

Radiation Deficient 51 (RAD51) foci are discrete nuclear puncta formed by the RAD51 recombinase at sites of DNA damage and replication stress. Because RAD51 loading onto resected DNA is a critical step in HR, RAD51 provides a functional readout of a cell’s ability to execute HR-mediated repair. Accordingly, abundant RAD51 foci in proliferating cells are consistent with functional HR, whereas low or absent foci support HRD observed in many tumors harboring BRCA1/2 alterations, and can also indicate HR restoration under therapeutic pressure (Fig. 2) [79,86,87]. DNA-based HRD classifiers can be clinically useful, but HR capacity is dynamic and may change during treatment. RAD51 therefore offers a functional, real-time measure of current HR competence, with potential utility both for refining patient selection for PARP inhibitors and for interpreting resistance at progression [86,87]. RAD51 assays quantify foci in tumor cells, typically restricting scoring to S/G2-phase cells using co-staining with a cell-cycle marker.

**Figure 2:**
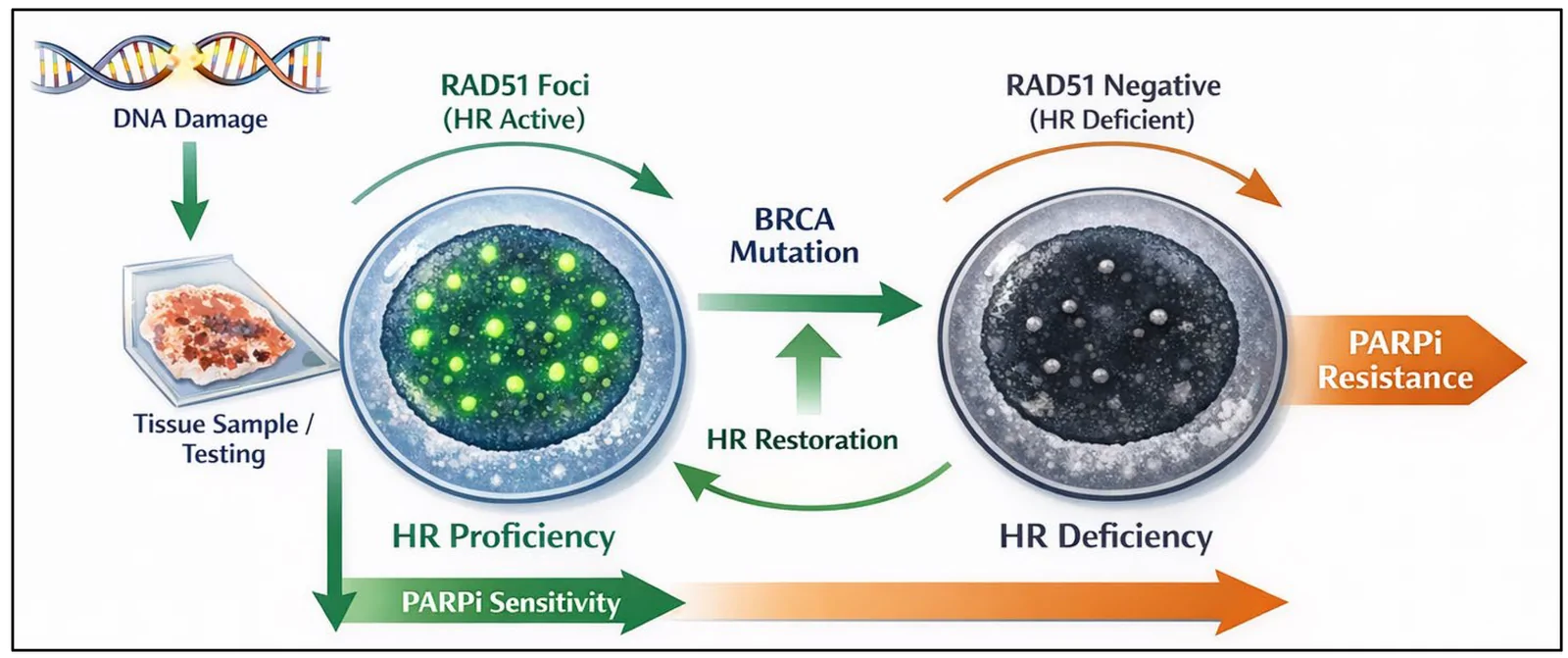
Biological rationale and clinical relevance of RAD51 foci assays. RAD51: Radiation Deficient 51; BRCA: Breast Cancer gene; HR: Homologous recombination; PARPi: Poly (ADP-ribose) polymerase inhibitor.

Schematic illustrating RAD51 foci as a functional readout of homologous recombination (HR) competency. Following DNA damage/replication stress, HR-proficient tumor cells show robust RAD51 nuclear foci formation (RAD51-positive), consistent with functional HR. In contrast, HR-deficient states, classically observed in many BRCA1/2-altered tumors, are characterized by low or absent RAD51 foci (RAD51-negative). HR restoration under therapeutic pressure is conceptually mirrored by re-emergence of RAD51 foci and is associated with reduced likelihood of benefit from PARP inhibitor rechallenge.

#### 6.2.2 Clinical Evidence

A key strength of RAD51 foci is that they can serve as a unifying functional endpoint across heterogeneous genetic resistance mechanisms. In germline BRCA-mutated breast cancer, RAD51 foci positivity has been associated with primary or acquired PARP inhibitor resistance, consistent with functional HR restoration even when a tumor retains a BRCA alteration label [72]. In broader clinical tumor sampling, a RAD51 assay feasible in routine specimens has identified HR-deficient states and predicted PARP inhibitor response beyond BRCA genotype, supporting RAD51 as an enrichment biomarker across BRCA-related contexts [86]. In ovarian cancer, early *ex vivo* irradiation-induced RAD51 assays demonstrated that RAD51 foci formation can stratify HR proficiency and predict PARP inhibitor response in translational settings [88].

#### 6.2.3 Practical Implementation

**Baseline use: resolving equivocal or discordant HRD signals**

In clinical and translational workflows, RAD51 foci testing adds the most value when the key question is current HR competence, rather than historical evidence of HRD. At baseline, RAD51 can refine stratification when DNA-based biomarkers are equivocal or clinically discordant, for example, when a tumor is BRCA-wildtype but behaves like HR-deficient, or when a BRCA-altered tumor shows unexpectedly limited sensitivity to platinum and/or PARP inhibitors. In these settings, a functional readout can supplement genotype-based classification and provide a more direct estimation of whether the HR pathway is operational in proliferating tumor cells.

**Post-treatment use: recurrence/progression and PARP inhibitor rechallenge decisions**

After exposure to platinum agents and/or PARP inhibitors, the interpretive weight of RAD51 increases at recurrence or progression because the clinical decision hinges on whether the tumor has functionally restored HR. In this context, a fresh biopsy is preferred, and RAD51-formalin-fixed paraffin-embedded (FFPE) testing can identify HR-proficient or HR-restored states that make PARP inhibitor rechallenge biologically implausible, even if the tumor is labelled as HRD-positive by other classifiers [72,74,75,76]. Conversely, a RAD51-low result indicates continued HR dysfunction and strengthens the rationale for PARP inhibitor sensitivity or PARP inhibitor-based combinations [72,89,90,91]. The most informative use of RAD51 is often integrated with other biomarkers (e.g., ctDNA evidence of evolving resistance genotypes) and applied within trial designs that test whether RAD51-defined HR status predicts benefit from specific DDR inhibitor strategies [89,91,92].

**Practical limitations and sources of variability**

Implementation of RAD51 foci testing in routine clinical practice remains technically challenging and unstandardized [86,89]. Accurate identification of S/G2-phase tumor cells requires co-staining with proliferation markers like geminin, introducing variability in protocols and scoring thresholds [86,89]. RAD51 foci assessment is semi-quantitative and subject to inter-observer variability, particularly in borderline cases [91]. Reproducibility is further affected by differences in image interpretation, field selection, and cut-off definitions for HR proficiency versus deficiency [90,91]. Antibody performance issues include specificity, cross-reactivity, and signal-to-noise ratio in formalin-fixed paraffin-embedded (FFPE) tissues [86,89]. Pre-analytical factors such as cold ischemia time, fixation duration, and tissue processing significantly impact antigen preservation and foci detectability, often leading to false-negative or inconclusive results, especially in real-world pathology workflows where strict standardization is difficult [86,89,90,91].

**Toward routine implementation: standardization and validation needs**

Collectively, these technical and methodological constraints currently limit the scalability and routine clinical integration of RAD51 foci assays [87,90]. Addressing these challenges will require assay harmonization, robust external validation, and the development of standardized scoring frameworks before widespread clinical adoption can be realized.

**Translational implications for DDR-combination strategies**

From a translational perspective, functional HR assessment using RAD51 foci may also have implications for patient selection in DDR-targeted combination strategies. RAD51-defined HR proficiency could help identify tumors less likely to benefit from PARP inhibitor monotherapy, but potentially susceptible to approaches targeting replication stress or checkpoint dependence, such as ATR, CHK1, or WEE1 inhibitor–based combinations. Conversely, RAD51-low tumors may represent contexts in which PARP inhibitor sensitivity is maintained and combination strategies can be rationally layered to enhance or prolong response. These considerations highlight the potential role of RAD51 not only as a biomarker of current HR status, but also as a tool to inform therapeutic positioning within an evolving DDR treatment landscape.

### 6.3 Genomic Scars and Mutational Signatures

#### 6.3.1 Rationale

Genomic scars and mutational signatures indicate HRD by identifying the accumulated footprints of impaired HRR at the DNA level. Unlike functional assays (RAD51 foci), which reflect current HR competence, scars and signatures encode the historical consequences of sustained HR dysfunction across many cell cycles. This perspective is clinically useful at baseline because HRD can arise through diverse mechanisms beyond BRCA1/2 mutations while still producing characteristic genomic patterns [93,94].

The well-established genomic scar framework quantifies chromosomal aberrations prevalent in HRD tumors. These aberrations include loss of heterozygosity (LOH), large-scale state transitions (LST), and telomeric allelic imbalance (TAI). They are often combined into an HRD score (LOH + LST + TAI), which has prespecified cutoffs originally tuned on BRCA1/2-deficient tumors (Fig. 3) [95]. These copy-number-based measures can be derived from SNP arrays or reconstructed from next-generation sequencing (NGS) data using computational implementations, enabling broader adoption across sequencing modalities [95,96]. Composite classifiers integrate multiple features to improve detection and generalizability [97,98].

**Figure 3:**
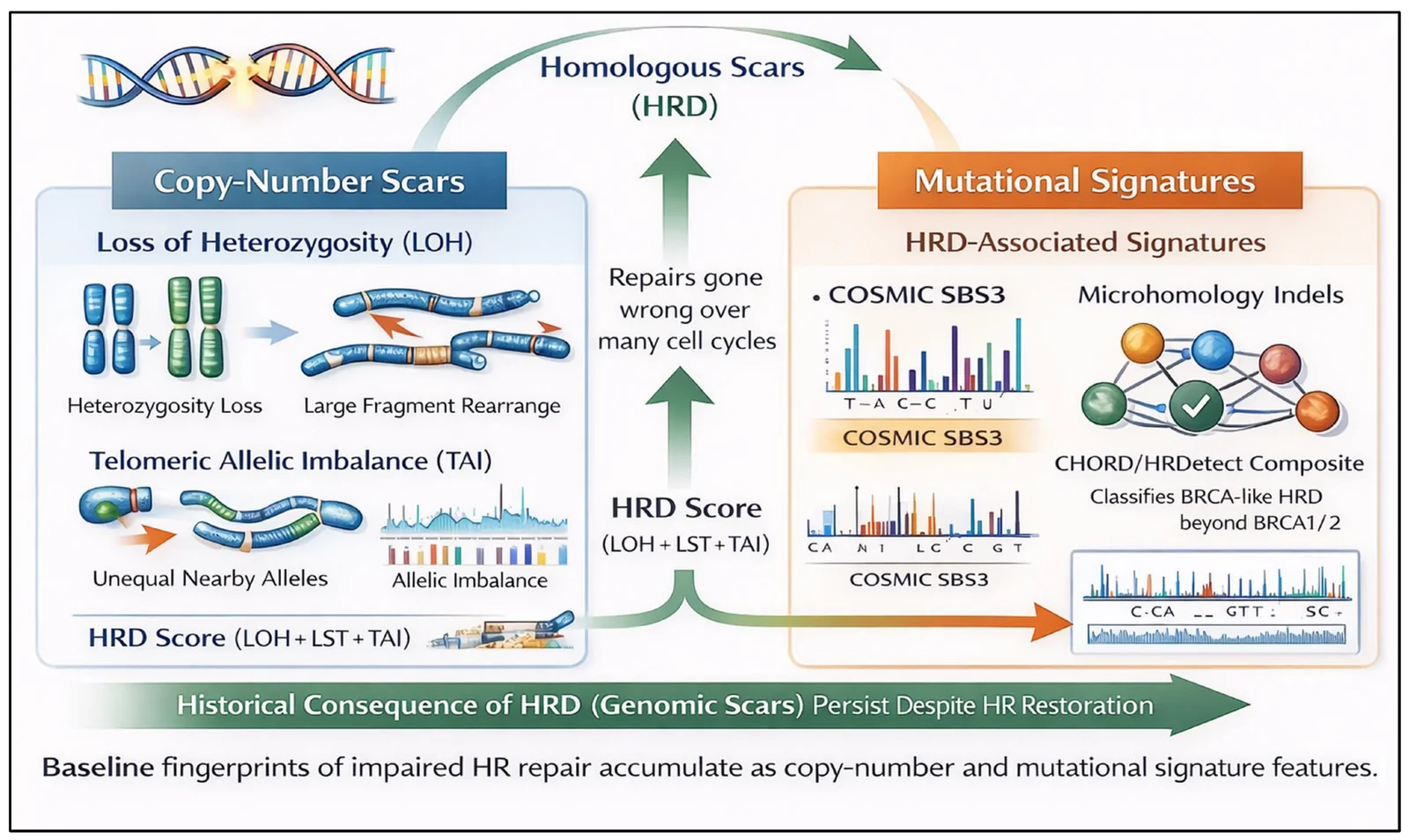
Genomic scars and mutational signatures as historical readouts of homologous recombination deficiency (HRD).

Schematic illustrating genomic scars and mutational signatures as “historical” readouts of homologous recombination deficiency (HRD). By capturing accumulated DNA-level footprints of impaired homologous recombination repair (HRR) across multiple cell cycles, these measures reflect the long-term consequences of sustained HR dysfunction, in contrast to functional assays that report current HR competence (e.g., RAD51 foci). HRD can arise through mechanisms beyond BRCA1/2 alterations yet converge on characteristic scar/signature patterns.

#### 6.3.2 Practical Implications

**Baseline use and scope (broad HRD discovery beyond *BRCA*)**

Genomic scars and signatures are best suited for baseline stratification and broad HRD discovery, including identification of HRD phenotypes beyond BRCA mutations.

**Temporal persistence limits dynamic monitoring (post-treatment HR restoration)**

One central limitation is temporal persistence: genomic scars are durable. Once established, LOH/LST/TAI patterns may remain detectable even if HR function is restored later, especially after exposure to platinum agents or PARP inhibitors [99,100,101]. Therefore, these assays are most informative for patient selection prior to initial DDR inhibitor exposure rather than for monitoring acquired resistance.

**Cross-platform and computational challenges in HRD score implementation**

Genomic scar scores (combining LOH, LST, and TAI) face substantial computational challenges across NGS platforms. First, platform-specific variability in coverage depth and fragment size impacts LOH detection, particularly with sparse single nucleotide polymorphism (SNP) coverage in targeted panels versus whole-genome sequencing. Second, large-scale state transitions (LSTs) defined as chromosomal breaks ≥ 10 megabases are sensitive to segmentation parameters and background noise; normalization strategies from SNP arrays may not transfer to low-pass whole-genome sequencing or targeted capture data. Third, telomeric allele imbalance (TAI) scoring requires copy number enumeration to chromosome ends, often compromised by poor subtelomeric coverage inherent to short-read sequencing. Fourth, composite HRD scores assume independence of LOH, LST, and TAI, but these metrics are not statistically independent; linear summation may overcount correlated events. Finally, batch effects and reference population differences introduce systematic biases across platforms. HRD scores should be interpreted as platform-relative rather than absolute quantities, and cross-platform comparability requires explicit analytical validation prior to clinical deployment.

**Spatial and temporal heterogeneity: sampling bias in advanced disease**

An additional limitation of genomic scar–based approaches relate to spatial and temporal tumor heterogeneity. Because these assays are typically derived from a single biopsy, they may not capture the full spectrum of clonal diversity present across primary and metastatic sites. Intratumoral heterogeneity and clonal branching can lead to coexistence of HR-deficient and HR-proficient subclones within the same patient, particularly in advanced disease. As a result, genomic scar measurements may be disproportionately influenced by the sampled lesion and may not reflect the dominant or treatment-relevant biology across all tumor compartments [94,99,100,101]. This limitation is further compounded over time, as selective pressures from platinum agents or PARP inhibitors can reshape clonal architecture, promoting expansion of HR-restored subclones that are not adequately represented by historical genomic scars. Consequently, static DNA-based HRD assays are inherently susceptible to sampling bias and may misclassify tumors in the context of evolving metastatic disease [102].

**Summary: strong for baseline, weaker for dynamic HR status**

Collectively, these limitations highlight that genomic scar–based assays, while robust for baseline stratification, have reduced reliability for capturing dynamic HR status in advanced and treatment-exposed settings.

### 6.4 Imaging Biomarkers and Dynamic Monitoring

#### 6.4.1 Rationale

Imaging biomarkers provide a non-invasive, repeatable method for capturing spatial heterogeneity and whole-body disease biology, addressing two key limitations of single-site tissue assays. In the DDR/HRD context, imaging can contribute at three distinct levels: (1) radiomics/radiogenomics, extracting quantitative features from routine CT, MRI, and PET scans to infer molecular phenotypes such as BRCA- and HRD-related states; (2) computational pathology, using AI-derived biomarkers from histology as a scalable, imaging-like surrogate of HRD; and (3) molecular imaging, employing PET tracers that quantify PARP1 expression and/or drug-target engagement *in vivo* (Fig. 4) [103,104,105,106,107].

**Figure 4:**
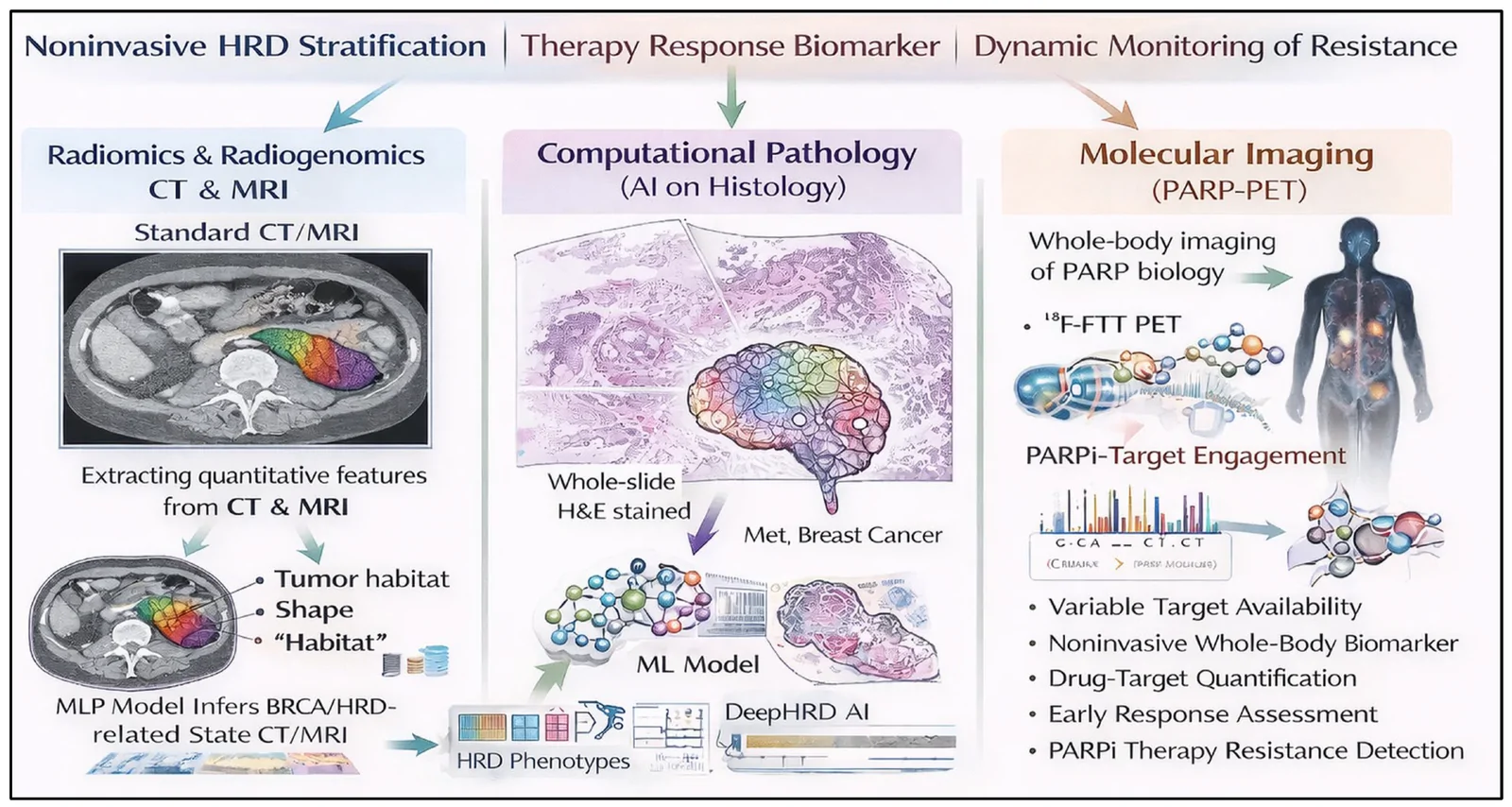
Imaging biomarkers as noninvasive, repeatable modalities to infer homologous recombination deficiency (HRD) and predict poly (ADP-ribose) polymerase (PARP) inhibitor benefit.

Schematic overview of imaging-based approaches that capture spatial heterogeneity and whole-body disease biology, addressing key limitations of single-site tissue assays. Three complementary layers are depicted: (1) radiomics/radiogenomics, which extracts quantitative features from routine CT/MRI/PET to infer molecular phenotypes including BRCA- and HRD-associated states; (2) computational pathology, where AI-derived features from digitized histology act as a scalable, imaging-like surrogate of HRD; and (3) molecular imaging, using PET tracers to quantify PARP1 expression and/or *in vivo* target engagement. Collectively, these modalities aim to improve baseline stratification and monitoring to identify patients most likely to benefit from PARP inhibitors (PARPi).

#### 6.4.2 Modalities and Evidence Base

***Radiomics/Radiogenomics:*** In ovarian cancer, these disciplines have advanced from proof-of-concept prognostic modelling to biologically anchored questions, such as predicting BRCA/HRD-related phenotypes from CT-derived texture and habitat features [103]. Studies have revealed associations between CT texture-derived tumor heterogeneity and, more recently, CT-based radiomics models have been reported to predict HRD score/status in ovarian cancer [104,105].

***Computational Pathology:*** Deep learning on routine histological slides has emerged as a scalable approach to inferring HRD phenotypes. In multicohort studies of breast and ovarian cancer, DeepHRD predicted HRD from whole-slide images and associated AI-inferred HRD with clinical outcomes [106]. Although not true radiologic imaging, this modality belongs to the category of “imaging biomarkers” because it transforms a visual phenotype into a deployable, repeatable classifier that can complement genomic HRD testing when access to sequencing or tissue quantity is limited.

***Molecular Imaging (PARP-PET):*** This approach uses PET imaging to quantify PARP1 expression and/or PARP inhibitor drug-target engagement across all lesions, addressing intrapatient heterogeneity and bypassing sampling limitations. Early translational clinical work demonstrated the feasibility of PARP1 PET imaging using [^18^F] FluorThanatrace (FTT) and revealed significant variability in uptake across lesions, consistent with variable target availability in ovarian cancer [107]. A pilot ovarian cancer study directly examined PARP inhibitor drug-target engagement with [^18^F] FTT PET as a biomarker of response. Parallel clinical studies in breast cancer suggested association between [^18^F] FTT uptake and response to PARP inhibitors [108]. First-in-human experiences with radiolabeled PARP inhibitors ([^18^F]-olaparib) provide further support for PARP-targeted imaging as a platform for patient selection and dynamic monitoring [109,110]. Prospective studies of broader populations with solid tumors and DDR alterations suggest that baseline [^18^F] FTT uptake varies depending on tumor type and treatment history [111].

#### 6.4.3 Practical Implementation

Imaging biomarkers, including radiomics and artificial intelligence (AI), can provide low-friction triage signals from standard imaging, while PARP-positron emission tomography (PARP-PET) offers mechanistically anchored readouts of target engagement [112]. However, the field faces substantial barriers to routine clinical implementation. Radiomics and AI remain highly sensitive to methodological variability in acquisition, reconstruction, and feature extraction, limiting reproducibility and cross-center generalizability; most models lack robust external validation [103,112]. For PARP-PET, practical constraints include limited radiotracer availability, specialized infrastructure needs, high costs, and insufficiently defined clinically actionable thresholds [107,111]. Additionally, evidence linking imaging readouts to treatment selection remains emerging. Consequently, imaging biomarkers are not yet ready for routine integration into DDR-targeted treatment algorithms and are currently best positioned as exploratory or complementary tools within prospective clinical trials [107,111,112]. A conceptual overview linking key biomarker modalities to their potential roles in therapeutic stratification and combination strategy selection is provided in Table 1.

**Table 1:** Biomarker–strategy alignment in DNA damage response (DDR)-targeted therapies.

Biomarker	Biological Readout	Clinical Implication	Relevant Therapeutic Strategies
**RAD51 foci**	Functional HR status (current)	Distinguishes HR-proficient vs. HR-deficient tumors at time of treatment	PARP inhibitor monotherapy vs. ATR/CHK1/WEE1-based combinations
**ctDNA (*BRCA* reversion)**	Emerging resistance/HR restoration	Identifies acquired resistance and dynamic clonal evolution	PARP inhibitor rechallenge vs. alternative DDR combinations
**Genomic scars (LOH/LST/TAI)**	Historical HRD	Baseline stratification	PARP inhibitor eligibility
**Imaging biomarkers (radiomics, PARP-PET)**	Spatial heterogeneity/target engagement	Whole-body assessment and dynamic monitoring	Treatment selection refinement and response monitoring

Abb.: HR—Homologous recombination, ATR—Ataxia telangiectasia and Rad3-related, CHK1—checkpoint kinase 1, BRCA—Breast cancer, PARP—Poly (ADP-ribose) polymerase, DDR—DNA damage response, LOH—Loss of heterozygosity, LST—Large-scale state transitions, TAI—telomeric allelic imbalance, HRD—homologous recombination deficiency.

### 6.5 Speckle-Type POZ Protein and Microsatellite Instability Alterations in Prostate Cancer

The immunological landscape of prostate cancer presents distinct challenges and opportunities for combining DDR inhibitors with immune checkpoint blockade. Speckle-type POZ protein (SPOP), a critical component of E3 ubiquitin ligase, is involved in the degradation of several proteins involved in regulating cellular growth. Microsatellite instability (MSI) results in products from the DNA mismatch repair mechanism. Recent studies from prostate cancer revealed that SPOP mutations and MSI status as contributing factors in determining the expression of PD-L1, consequently, the responding potential for PD-1 therapy [113]. SPOP and MSI assessment represent potential markers for selecting patients with prostate cancer who are more likely to respond to immunotherapy or to combined treatment.

### 6.6 Pre-Analytical Variables and Site-Specific Discordance in Biomarker Assessment

The anatomical source of biopsy profoundly influences DDR deficiency and resistance biomarkers. Primary tumors and metastatic lesions exhibit substantial discordance in HRD-related genomic scars, BRCA mutation status, and RAD51 scores, with reported rates of 15–40% across solid tumors. This spatial heterogeneity means a single-site biopsy may misrepresent dominant resistance mechanisms. Bone metastases present unique challenges. Decalcification protocols, especially formic acid-based methods, compromise nucleic acid integrity through DNA fragmentation and crosslinking, making specimens unsuitable for comprehensive genomic profiling. Even EDTA-based decalcification introduces variability without standardization across laboratories. Consequently, patients with predominant bone metastatic disease (common in prostate and breast cancers) may be systematically under-represented in biomarker-enriched trials or assigned incorrect HRD status. Alternative approaches include: (1) obtaining concurrent soft tissue metastases when feasible; (2) prioritizing plasma ctDNA analysis in bone-predominant disease; and (3) developing validated decalcification protocols with routine quality control metrics. These pre-analytical considerations should be explicitly documented in clinical studies reporting biomarker discordance.

## 7 Combination Strategies to Forestall or Overcome Resistance

Resistance to DDR inhibitors, particularly PARP inhibitors, most commonly reflects restoration of DNA repair capacity (including partial or complete HR recovery) and/or adaptive tolerance of replication stress. Combination therapeutic strategies therefore target four complementary objectives: (1) disabling compensatory stress-response circuitry, (2) leveraging DNA damage-driven immunogenic signaling, (3) improving tumor selectivity via targeted delivery or induced protein degradation, and (4) optimizing dosing and sequencing to maximize synthetic lethality [114,115]. The effective implementation of these combination strategies is closely linked to biomarker-driven patient selection, including functional assays (RAD51 foci), dynamic monitoring approaches (ctDNA), and genomic HRD classifiers, which together may help define context-specific therapeutic vulnerabilities.

### 7.1 Dual Inhibition of DDR Kinases

#### 7.1.1 Rationale

Dual DDR kinase inhibition is based on the premise that tumors adapting to DDR inhibitors, especially after PARP inhibitor exposure, may become dependent on replication stress signaling and cell cycle checkpoint control to prevent replication-associated lesions from becoming lethal chromosome damage. Upstream DDR kinases (ATM, ATR, DNA-PK) integrate lesion recognition with repair pathway deployment, whereas ATR-CHK1 signaling and WEE1 functionally enforce replication-stress tolerance by regulating origin firing, fork stability, and sustaining intra-S and G2 checkpoint control [116].

Among the most clinically emphasized strategies, the combination of ATR inhibition and PARP inhibition is the most mature in terms of both mechanism and translation. PARP inhibitors increase replication-associated lesions and fork stress, while ATR inhibitors suppress tolerance and stabilization of replication forks. In ovarian cancer models, the combination of PARP and ATR inhibition has been shown to overcome acquired resistance to PARP inhibitors and platinum agents with antitumor activity [117]. Related approaches include CHK1 inhibitor + PARP inhibitor and WEE1 inhibitor + PARP inhibitor, which share the objective of converting checkpoint dependence into replication catastrophe [114,115,116].

#### 7.1.2 Combination Regimens

Dual DDR-kinase approaches can be conceptualized as modular strategies that pair a DDR stressor (often a PARP inhibitor) with checkpoint or repair kinase inhibition to prevent compensatory rescue (Fig. 5). Multiple combination regimens are currently being evaluated in ongoing early-phase trials; however, for many approaches the clinical evidence base remains immature, with limited published efficacy data (Table 2).

**Figure 5:**
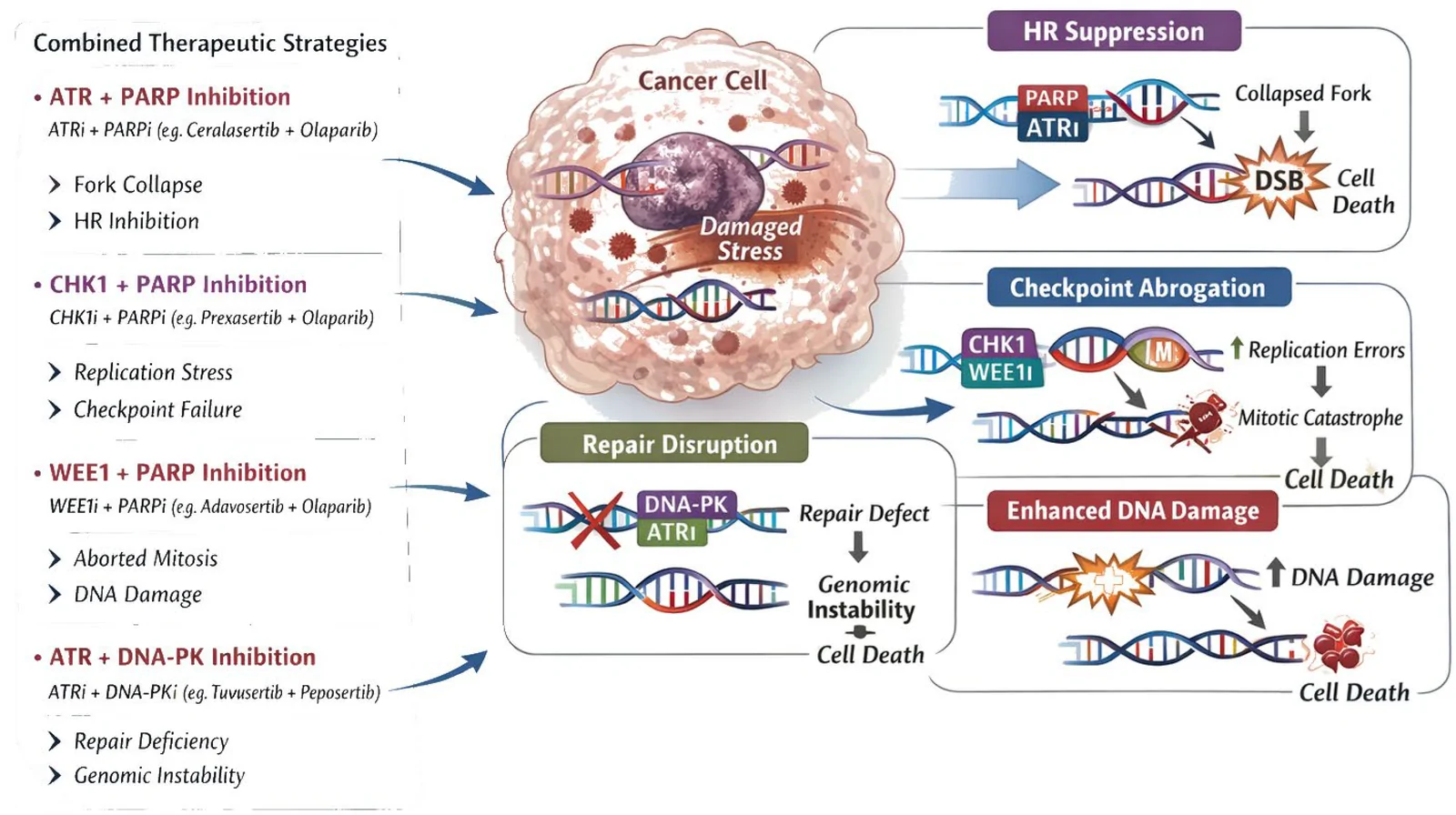
Schematic overview of dual DNA damage response (DDR) inhibition strategies designed to overcome resistance to PARP inhibition.

Schematic illustrating representative combination modules, ATR inhibitor (ATRi) + PARP inhibitor (PARPi), CHK1 inhibitor (CHK1i) + PARPi, WEE1 inhibitor (WEE1i) + PARPi, and ATRi + DNA-PK inhibitor (DNA-PKi), and the principal mechanistic outcomes proposed to drive antitumor activity. ATRi + PARPi is depicted as amplifying replication stress and fork instability, promoting fork collapse and conversion of replication-associated lesions into double-strand breaks (DSBs). CHK1i + PARPi and WEE1i + PARPi are shown to impair checkpoint signaling and CDK control, increasing replication errors and triggering mitotic catastrophe. ATRi + DNA-PKi is shown to disrupt compensatory repair by concurrently inhibiting the replication-stress response and end-joining repair, thereby promoting genomic instability and cell death.

**Table 2:** Details of ongoing clinical trials assessing the combination regimens.

Strategy	Agents	Trial/ID	Phase	Population (Key)	Regimen (High Level)	Key Reported Outcomes (Early)	Ref.
ATR inhibitor + PARP inhibitor	ceralasertib + olaparib	CAPRI (NCT03462342)	II (signal-finding)	Recurrent platinum-sensitive high grade serous ovarian cancer (HGSOC); BRCA1/2 mut or HRD; acquired PARP inhibitor resistance (benefit → progressed on PARP inhibitor as penultimate regimen)	ceralasertib 160 mg D1–7 + olaparib 300 mg BID continuous (28-day cycles)	ORR 50% (6/12 PR; 95% CI 0.15–0.72); median Progression free survival (PFS) 7.43 mo; grade ≥ 3 toxicities primarily hematologic	[118]
CHK1 inhibitor + PARP inhibitor	prexasertib + olaparib	(no short name in paper)	I (3 + 3 + expansion)	HGSOC + other solid tumors; key subset: BRCA1-mut, PARP inhibitor-resistant HGSOC	olaparib lead-in; then prexasertib D1 & D15 + attenuated olaparib (intermittent)	In BRCA1-mut PARP inhibitor-resistant HGSOC: 4/18 partial remission (PR); common trial-related adverse events (TRAEs): leukopenia/neutropenia/thrombocytopenia/anemia; PD biopsies: decreased RAD51 foci, increased γH2AX/pKAP1/pRPA	[119]
WEE1 inhibitor + PARP inhibitor	adavosertib + olaparib	NCT02511795	Ib (dose-finding + expansion)	Refractory solid tumors (Part A); plus SCLC expansion (Part B)	adavosertib intermittent schedules + olaparib (continuous in RP2D)	Part A: objective response rate (ORR) 14.8% (95% CI 8.7–22.9) overall; limited antitumor activity; common TRAEs: fatigue/diarrhea/nausea/anemia	[64]
ATR inhibitor + PARP inhibitor	camonsertib + niraparib/olaparib	NCT04972110	I/II	Advanced solid tumors (biomarker-enriched in program)	Combination cohorts (intermittent designs typical for ATR inhibitors)	No peer-reviewed efficacy results required for table (registry lists design; public readouts may exist as abstracts/press)	[120]
ATR inhibitor ± PARP inhibitor/ICI	ceralasertib ± olaparib ± durvalumab	NCT03682289	II	Solid tumors (multiple cohorts)	Multi-arm	Registry describes arms; peer-reviewed combo outcomes not required for table	[121]
ATRinhibitor + PARP inhibitor	elimusertib + niraparib	NCT04267939	I/II	Advanced solid tumors/ovarian cancer	Combination	Registry describes; results not mandatory for table	[122]
DNA-PKcs inhibitor + ATR inhibitor	peposertib + tuvusertib	NCT05687136	I	Advanced/metastatic solid tumors	Combination	Registry describes; results not mandatory for table	[123]

***ATRi + PARP inhibitor:*** Consistent with re-sensitization, clinical activity of ATRi + PARP inhibitor combinations has been reported in acquired PARP inhibitor-resistant, HR-deficient ovarian cancer, as demonstrated in the phase II CAPRI trial evaluating ceralasertib plus olaparib [45]. Ongoing studies include camonsertib (RP-3500) plus niraparib or olaparib (NCT04972110), ceralasertib (AZD6738) alone or with olaparib (and/or durvalumab) (NCT03682289), and elimusertib (BAY1895344) plus niraparib (NCT04267939).

***CHK1i + PARP inhibitor and WEE1i + PARP inhibitor:*** A phase I study of prexasertib (CHK1i) plus olaparib demonstrated preliminary clinical activity in BRCA-mutant HGSOC that had previously progressed on PARP inhibitors, accompanied by pharmacodynamic evidence consistent with compromised HR function and increased DNA damage/replication stress markers [119]. For WEE1, adavosertib plus olaparib has been evaluated in phase Ib trial in refractory solid tumors [64].

***Dual DDR Kinase Blockade of Repair and Checkpoint Pathways:*** A more direct dual-kinase strategy pairs distinct DDR kinases to block compensatory pathways. For example, peposertib (DNA-PK inhibitor) combined with tuvusertib (ATR inhibitor) is being tested in a phase I study (NCT05687136) [123].

While efficacy signals such as the 50% objective response rate with ceralasertib plus olaparib in the CAPRI trial [118] are encouraging for acquired PARP inhibitor resistance, a critical risk-benefit assessment is necessary. Hematologic toxicity is the primary dose-limiting constraint, with grade 3 or higher adverse events (neutropenia, thrombocytopenia, leukopenia, anemia) exceeding 40–60%, higher than PARP inhibitor monotherapy due to overlapping myelosuppressive effects on hematopoietic progenitors. The CAPRI trial’s 50% response rate was achieved in a highly selected, platinum-sensitive, BRCA-mutant/HRD population, with median progression-free survival of ~7.4 months, weighed against significant dose reductions or discontinuations due to cytopenias. Prexasertib (CHK1 inhibitor) plus olaparib showed activity in a small subset but with frequent grade 3/4 hematologic events [119]. Adavosertib (WEE1 inhibitor) plus olaparib yielded a modest 14.8% response rate with notable fatigue, diarrhea, nausea, and anemia [64]. These dual combinations are not ready for routine use outside specialized centers or trials. Optimal roles may be restricted to patients with documented acquired resistance, good performance status, adequate bone marrow reserve, and close monitoring. Schedule optimization (intermittent dosing) is rational but lacks comparative data. Until further evidence emerges, shared decision-making must address trade-offs between tumor response and substantial myelosuppression risk.

### 7.2 Combining DDR Inhibitors with Immunotherapy

#### 7.2.1 Rationale

The rationale for combining DDR inhibitors with immune checkpoint inhibitors (ICI) is that DNA damage and replication stress can act as an upstream immunologic stimulus, potentially converting immune-cold tumors into contexts more permissive for T-cell-mediated control. PARP inhibition and broader DDR disruption may increase cytosolic DNA fragments and micronuclei, thereby engaging innate DNA sensing (notably cGAS-STING) and type I interferon programs that support antigen presentation and T-cell priming [124,125,126]. In parallel, DDR perturbation can alter the tumor immunophenotype through effects on antigenicity and immune evasion, including modulation of PD-L1 expression and changes in inflammatory chemokine signaling (Fig. 6) [125,126,127]. In this context, established immunotherapy biomarkers such as tumor mutational burden (TMB) and MSI status may further modulate response to DDR inhibitor–ICI combinations. High TMB and MSI-high tumors are associated with increased neoantigen load and enhanced baseline immunogenicity, which may potentiate the immune-activating effects of DDR-induced DNA damage [124,125,126,128,129]. Conversely, in tumors with low TMB and microsatellite stability, DDR inhibition may act as a priming mechanism to augment immunogenicity, although the extent and durability of this effect remain variable [124,125,126,128,129]. These considerations suggest that integration of DDR-specific biomarkers with established immunotherapy markers may be necessary to refine patient selection for combination strategies.

**Figure 6:**
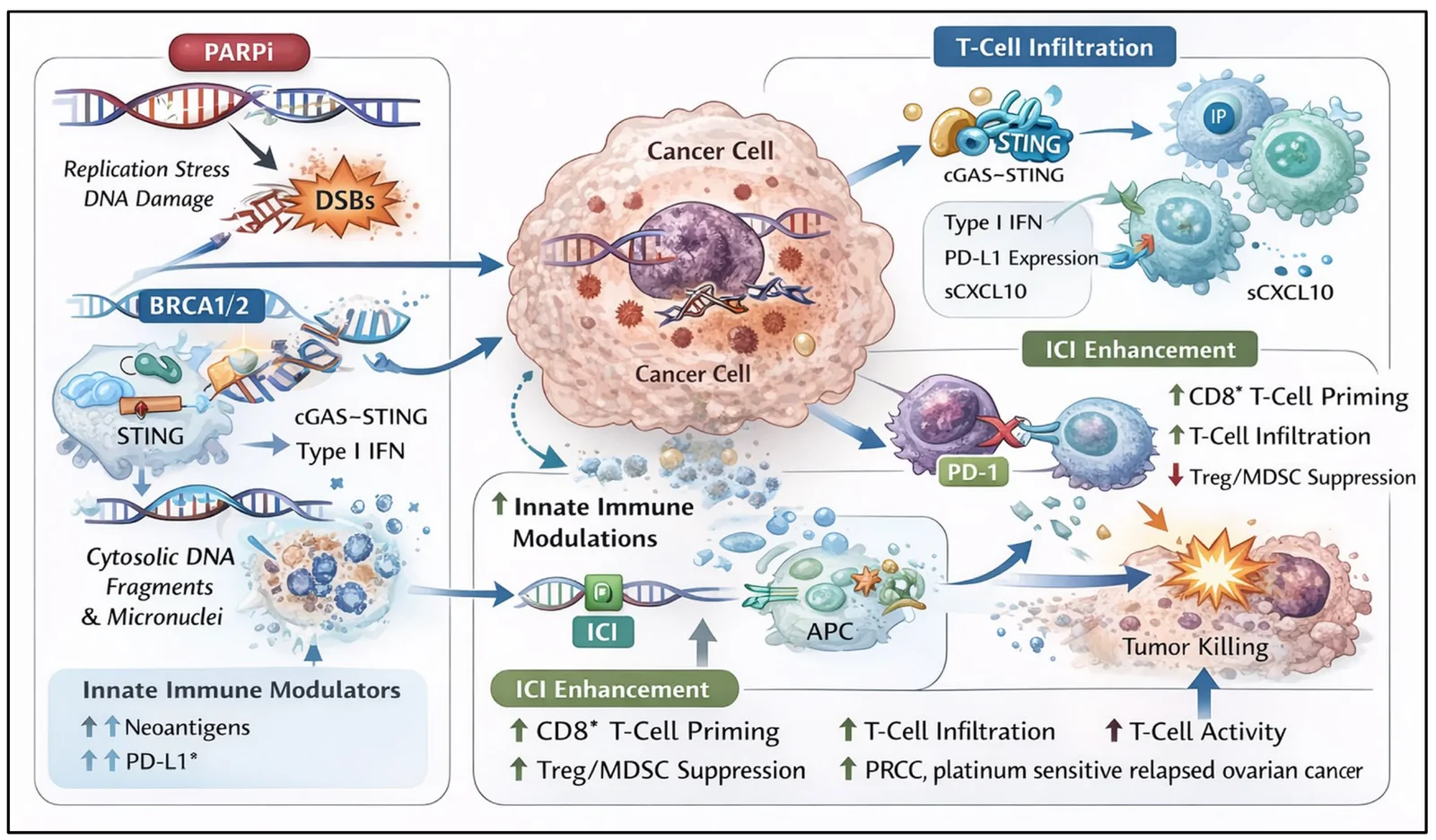
Mechanistic schematic of combining DNA damage response inhibitors (DDRi) with immune checkpoint inhibitors (ICI).

DDR inhibition (illustrated by PARP inhibition) increases replication stress and DNA damage, leading to cytosolic DNA fragments and micronuclei that activate innate DNA sensing (cGAS–STING) and downstream type I interferon signaling. This may enhance antigen presentation, induce pro-inflammatory chemokine programs, and promote immune cell recruitment; in parallel, DDR perturbation can induce adaptive immune resistance, including context-dependent PD-L1 upregulation. Immune checkpoint blockade (e.g., PD-1/PD-L1 inhibition) is depicted as restoring or augmenting effector T-cell activity within the tumor microenvironment, thereby potentiating tumor cell killing in DDR-perturbed tumors.

#### 7.2.2 Clinical Evidence

Early-phase clinical studies have demonstrated feasibility and provided mixed signals regarding efficacy, often suggesting that benefits are concentrated in specific molecular or tumor contexts rather than across unselected populations.

In recurrent ovarian cancer and triple negative breast cancer (TNBC), the TOPACIO/KEYNOTE-162 phase I/II trial evaluated niraparib plus pembrolizumab and reported promising antitumor activity [130]. A phase II study focusing on ovarian cancer reported modest clinical activity for olaparib plus durvalumab [131]. In a multi-tumor basket framework, the MEDIOLA phase II trial has explored olaparib plus durvalumab (with additional cohorts incorporating bevacizumab), providing longer-term signal [132]. The JAVELIN PARP Medley phase II evaluated avelumab plus talazoparib across tumor types and reported that overall response rates were broadly comparable to historical expectations for monotherapy [133].

Beyond metastatic settings, DDR inhibitor + immune checkpoint inhibitor (ICI) has also been tested in earlier-stage disease as part of multi-agent regimens. For instance, in the I-SPY2 trial focusing on high-risk HER2-negative breast cancer, adding durvalumab plus olaparib to paclitaxel increased pathological complete response rates in specific biomarker subsets, illustrating the potential of DDR-immune co-targeting in neoadjuvant strategies [134]. Several late-phase trials have sought to define clinical utility in frontline therapy. In advanced BRCA-non-mutated epithelial ovarian cancer, KEYLYNK-001 (pembrolizumab plus chemotherapy followed by maintenance pembrolizumab plus olaparib, with or without bevacizumab) has reported meeting a progression-free survival endpoint, with additional details awaited [135]. Key clinical trials evaluating DDR inhibitor–ICI combinations are summarized in Table 3.

**Table 3:** Ongoing registered clinical trials assessing DDRi–ICI combinations.

Strategy	DDRi + ICI	Study/ID	Phase	Population (Key)	Regimen (High Level)	Key Reported Outcomes (Early)	Status/Source
PARP inhibitor + PD-1	niraparib + pembrolizumab	TOPACIO/KEYNOTE-162/NCT02657889	I/II	Recurrent ovarian carcinoma (incl. platinum-resistant; irrespective of BRCA/HRD)	niraparib continuous + pembro q3w	Ovarian cohort (evaluable): ORR 18%, disease control rate (DCR) 65%; ORR broadly similar across BRCA/HRD/platinum subgroups	[130]
PARP inhibitor + PD-L1	olaparib + durvalumab	(no short name)	II	Recurrent ovarian cancer (predominantly platinum-resistant; majority BRCA-wt)	olaparib continuous + durvalumab q4w	ORR 14% (5/35); DCR 71%; correlative immune activation signals reported	[131]
PARP inhibitor + PD-L1	olaparib + durvalumab	MEDIOLA/NCT02734004	II (multi-cohort)	Platinum-sensitive relapsed ovarian cancer (PSROC), stratified cohorts	olaparib + durvalumab; selected cohorts added bevacizumab	gBRCAm PSROC doublet cohort: ORR 92.2%; non-gBRCAm cohorts: primary endpoint DCR at 24w 28.1% (doublet) vs. 74.2% (triplet incl. bevacizumab)	[132]
PARP inhibitor + PD-L1	talazoparib + avelumab	JAVELIN PARP Medley/NCT03330405	Ib/II	Multi-tumor, prospectively defined cohorts (incl. TNBC; DDR+ HR+/HER2-BC; BRCA-altered platinum-sensitive OC)	talazoparib daily + avelumab q2w	Phase 2 ORR: 18.2% (TNBC); 34.8% (DDR+ HR+/HER2-BC); 63.6% (platinum-sensitive BRCA-altered OC); hematologic ≥ G3 AEs common	[133]
PARP inhibitor + PD-L1 (neoadjuvant, triplet)	olaparib + durvalumab (with paclitaxel)	I-SPY2 (DOP arm)	Adaptive phase II	High-risk stage II/III HER2-negative breast cancer	durvalumab + olaparib added to neoadjuvant paclitaxel	pCR increased: all HER2-neg 20% → 37%; HR+/HER2-14% → 28%; TNBC 27% → 47%; MP2 subset HR+/HER2-: 64% vs. 22%	[134]
PARP inhibitor + PD-1 (frontline, maintenance strategy)	pembrolizumab + chemotherapy → maintenance pembrolizumab + olaparib (± bevacizumab)	ENGOT-OV43/GOG-3036/KEYLYNK-001/NCT03740165	III	Newly diagnosed advanced BRCA-nonmutated epithelial ovarian cancer	pembrolizumab + carboplatin/paclitaxel, then maintenance pembrolizumab + olaparib (design per protocol)	Public reports indicate PFS improvement for pembro + chemo → pembro + olaparib arm vs. control; full peer-reviewed detail still maturing across sources	[135]

### 7.3 Targeted Delivery and Proteolysis-Targeting Chimeras (PROTACs)

#### 7.3.1 Rationale

A recurring limitation of many DDR inhibitor combinations is dose-limiting hematologic toxicity. Two conceptually distinct but mechanistically aligned strategies to improve the therapeutic index are (1) targeted delivery and (2) targeted protein degradation [136,137]. In the DDR context, these approaches are particularly relevant for PARP pathway targeting, where efficacy may depend not only on catalytic inhibition but also on PARP1 trapping and broader chromatin-associated functions [138].

#### 7.3.2 Targeted Delivery Approaches

Targeted delivery strategies are being explored to optimize pharmacokinetics, tumor accumulation, and tolerability of DDR-directed regimens (Fig. 7). These strategies include nanoparticle-based formulations and tumor-targeted delivery of mechanistically complementary cytotoxic agents combined with DDR blockade. The rationale is to increase tumor-selective DNA damage, limit systemic exposure, and reduce repair-mediated escape [136,139].

**Figure 7:**
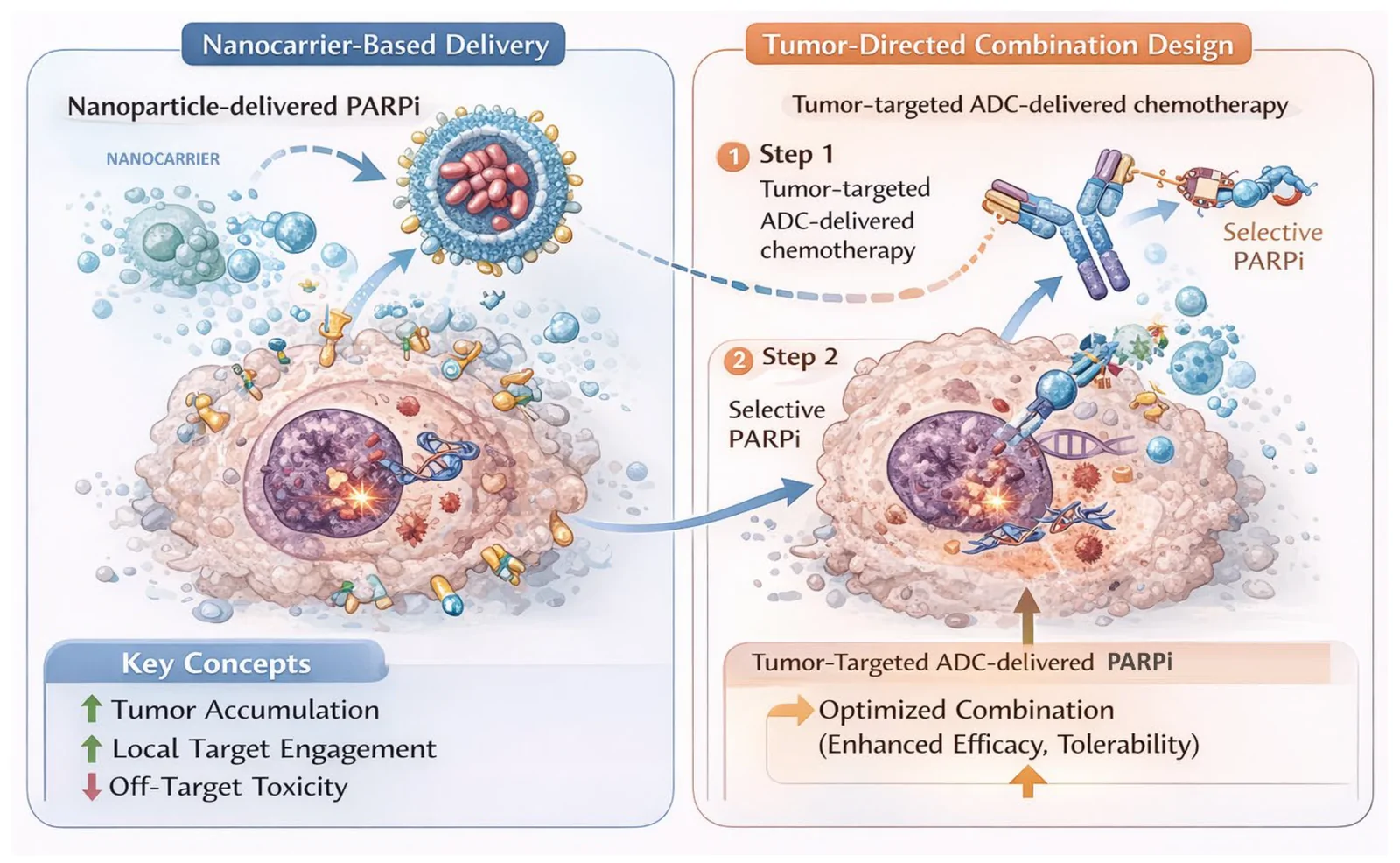
Targeted delivery approaches to enhance the therapeutic index of DDR inhibition.

Schematic illustrating two complementary design paradigms. Left: nanocarrier-based delivery of a DDR inhibitor (illustrated as PARP inhibition) to increase tumor accumulation and local target engagement while reducing systemic exposure and off-target toxicity. Right: tumor-directed combination architectures in which a tumor-targeted cytotoxic payload (illustrated as an antibody–drug conjugate [ADC]–delivered chemotherapy) concentrates DNA-damaging stress within tumor tissue, followed by a mechanistically matched DDR inhibitor (e.g., PARPi) to limit repair-mediated tolerance and enhance efficacy, with schedule and selectivity intended to improve tolerability.

One example is CRLX101, a nanoparticle formulation of a topoisomerase I (TOP1) inhibitor, evaluated in combination with olaparib in a phase I/II study (NCT02769962) [140]. This approach paired tumor-targeted TOP1 inhibitor delivery with schedule-optimized PARP inhibition. A related emerging strategy is the combination of tumor-targeted DNA-damaging payloads with DDR blockade. Examples include ADC-delivered TOP1 inhibitor payloads combined with next-generation PARP1-selective inhibitors. Although still early in development, preclinical and early translational data support this broader therapeutic concept [141,142].

#### 7.3.3 PROTACs and Targeted Protein Degradation

**Concept and rationale**

Rather than inhibiting the active site, PROTACs reduce target protein abundance, which can potentially suppress catalytic and scaffolding functions and enable durable pathway shutdown [137,143]. In oncology, PROTAC technology has progressed from concept to clinical trials for various targets, including DDR proteins [143,144].

**Preclinical PARP1 degrader examples and efforts to improve selectivity**

Several PARP1 degraders have been developed preclinically. For example, SK-575 was reported to be a potent PARP1 degrader that can induce PARP1 degradation and demonstrate antitumor efficacy in BRCA1/2-deficient models [145]. Additional work has addressed key translational constraints, such as tumor selectivity and linker optimization. For example, a tumor-selective PARP1 PROTAC strategy was developed with the explicit goal of improving the therapeutic window [146].

**Pharmacologic and pharmacokinetic constraints limiting translation**

PROTACs face substantial liabilities despite theoretical advantages over catalytic inhibition. First, their bifunctional architecture results in high molecular weights (>700 Da) exceeding “Rule of Five” parameters, challenging oral bioavailability [147]. Second, elevated topological polar surface area (tPSA, 150–200 Å^2^) due to multiple amide bonds and ionizable groups limits passive diffusion across lipid bilayers [148]. Combined high molecular weight and tPSA predispose PROTACs to efflux transporter substrates, reducing intracellular concentrations [149]. Third, the “hook effect” (high-concentration inactivation) produces a bell-shaped concentration–response curve where excessive dosing paradoxically reduces degradation efficacy, imposing narrow therapeutic windows and necessitating precise dose optimization [150,151]. Fourth, PROTACs exhibit event-driven pharmacology: degradation can persist after drug clearance, increasing risk of sustained off-tissue degradation and toxicity [152]. Additionally, requirement for functional ubiquitin-proteasome machinery means tumors with defective proteasome function may be intrinsically resistant. Translating PARP-targeting PROTACs to clinical therapies requires rational linker design, formulation strategies to enhance bioavailability and tumor penetration (e.g., nanoparticle encapsulation), and careful dose-finding studies to avoid the hook effect. The field would benefit from systematic pharmacokinetic–pharmacodynamic modeling to define achievable therapeutic windows before clinical trials.

**Anticipated resistance mechanisms specific to PROTAC biology (E3 ligase dependence)**

The dependency of PROTACs on specific E3 ubiquitin ligases [such as cereblon or Von Hippel–Lindau (VHL)] creates an inherent vulnerability: selective pressure will drive emergence of tumors that disable these ligases [153]. Anticipated resistance mechanisms include: (1) loss-of-function mutations or deletions in E3 ligase genes, abolishing ternary complex formation; (2) missense mutations within the PROTAC-binding domain of the ligase that disrupt degrader engagement while preserving native substrate recognition; (3) competitive upregulation of endogenous E3 ligase substrates that titrate away available ligase; and (4) mutations in the target protein at the PROTAC interface that prevent ternary complex assembly without abrogating catalytic function [154]. From a translational standpoint, this vulnerability could be mitigated by employing E3 ligases that are essential for tumor cell survival (limiting the feasibility of loss-of-function mutations) and sequential or alternating use of PROTACs engaging distinct E3 ligases to raise the evolutionary barrier [155]. Additionally, future clinical trials with PROTACs should consider integrating ctDNA-based monitoring for emerging E3 ligase mutations. Without proactive attention to these resistance routes, PROTACs risk recapitulating the same pattern of acquired resistance that has limited first-generation DDR inhibitors.

**Evidence gap and current clinical outlook**

Despite encouraging preclinical activity, PROTAC-based approaches targeting PARP1 and related DDR components have not yet demonstrated clinical efficacy in patients with PARP inhibitor–resistant disease. To date, available evidence remains largely limited to preclinical models, and early-phase clinical data are either lacking or still emerging. Consequently, the therapeutic potential of PROTAC-mediated degradation in this context should be considered exploratory, and expectations regarding clinical impact should be tempered until robust clinical validation becomes available.

### 7.4 Scheduling and Sequencing to Maximize Synthetic Lethality

#### 7.4.1 Rationale

For DDR inhibitor combinations, the limiting factor is not mechanistic plausibility but rather therapeutic index, most constrained by overlapping hematologic toxicity. Therefore, schedule design is the primary means of preserving sufficient target engagement in the tumor while allowing normal proliferative tissues to recover [114,116]. The goal of schedule optimization is to align drug exposure with the period when tumor cells are most vulnerable, which is usually when replication stress and DNA lesions are at their peak, while minimizing chronic suppression of repair and checkpoint pathways in normal tissues [114,116].

#### 7.4.2 Scheduling Patterns in DDRi Combinations

***Intermittent Checkpoint Inhibition with Continuous PARP Inhibition:*** A common pattern in ATR inhibitor + PARP inhibitor trials is continuous PARP inhibitor dosing with short ATR inhibitor exposure windows per cycle (e.g., days 1–7 of a 28-day cycle), reflecting the need to balance replication-stress intensification with marrow recovery [156,157]. Similar principles apply to WEE1 inhibitor and CHK1 inhibitor combinations, where intermittent schedules and attenuated PARP inhibitor dosing are frequently used (Fig. 8) [119].

**Figure 8:**
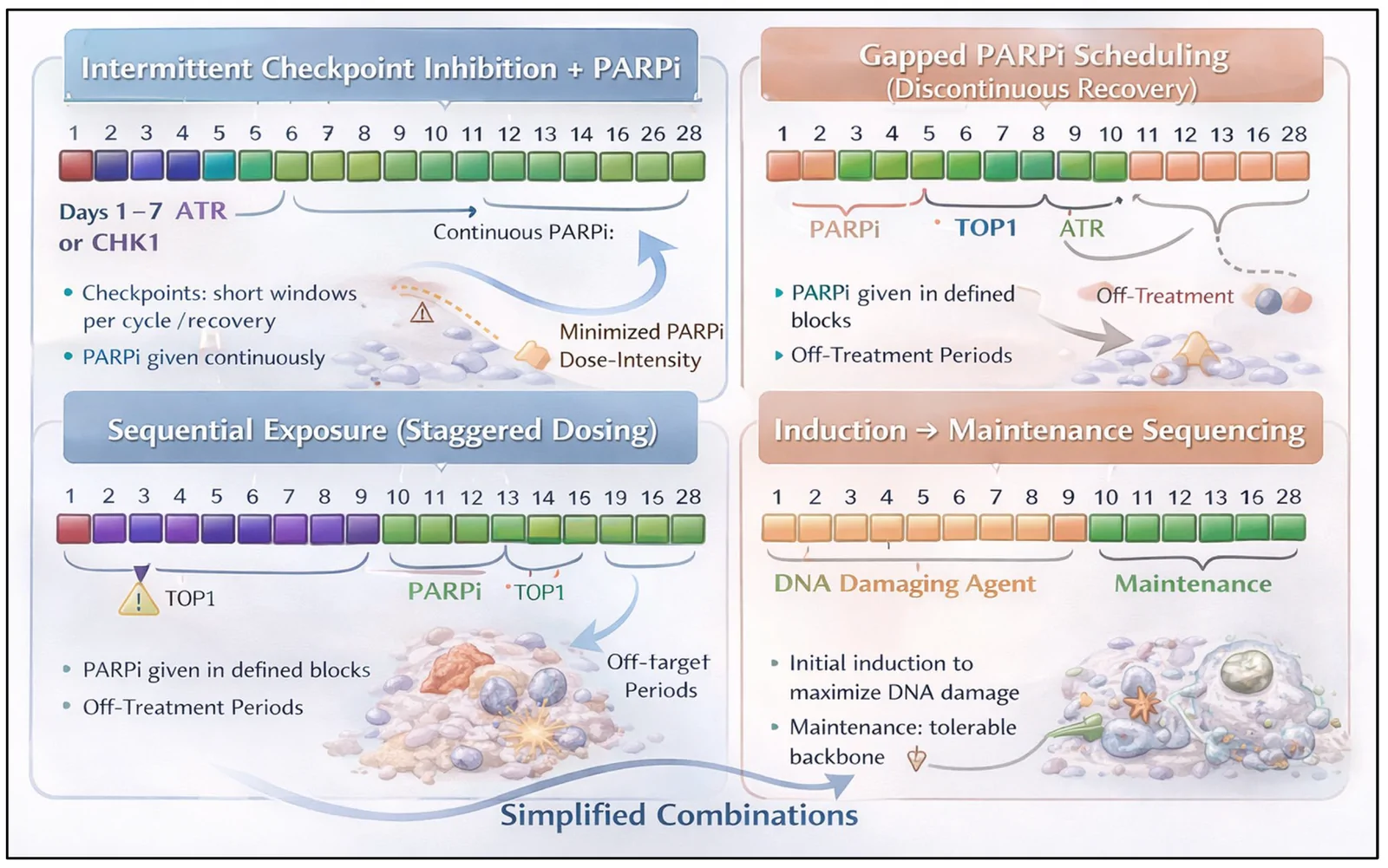
Scheduling patterns in DDR inhibitor (DDRi) combinations.

Schematic illustrating recurring dosing architectures used to maximize synthetic lethality while managing overlapping toxicities, particularly myelosuppression. Top left: intermittent checkpoint inhibition (e.g., ATRi/CHK1i/WEE1i administered in short windows within each cycle) layered onto continuous PARP inhibition (PARPi). Top right: ‘gapped’ PARP inhibitor scheduling, in which PARP inhibitor is delivered in defined blocks separated by planned off-treatment intervals to facilitate recovery and enable combination with additional DNA-damaging stressors. Bottom left: sequential (staggered) exposure, where agents are temporally ordered rather than fully concurrent to test whether antitumor activity can be retained with improved tolerability. Bottom right: induction–maintenance sequencing, in which an induction phase intensifies DNA damage or replication stress, followed by a maintenance backbone selected for sustained pressure and tolerability.

***Discontinuous PARP Inhibition:*** Combining PARP inhibitors with DNA-damaging agents (e.g., TOP1 inhibition) can lead to continuous exposure that is dose-prohibitive. One solution is gapped PARP inhibitor scheduling, in which the PARP inhibitor is administered in defined blocks separated by planned off-treatment intervals [140].

***Sequential Exposure:*** Preclinical studies suggest that sequential ATR and PARP inhibition can overcome acquired resistance in certain situations. The hypothesis is that sequential regimens may maintain antitumor activity while reducing dose-limiting toxicity [158].

***Induction–Maintenance Designs:*** In multi-agent regimens, sequencing can be employed to prime tumors with DNA damage or replication stress during an induction phase and subsequently sustain pressure on residual disease using a better-tolerated maintenance backbone therapy. This is particularly relevant for triplet regimens and for integrating ICIs [114,124].

## 8 Translational Outlook: from Bench to Bedside

### 8.1 Lessons from Clinical Trials: Successes and Failures

#### 8.1.1 Successes

In DDR therapeutics, mechanistic plausibility is rarely the main obstacle to success. Instead, translation often succeeds or fails at the intersection of dependency, deliverability, and design. PARP inhibitor maintenance studies in BRCA-mutant ovarian cancer demonstrate long-term survival benefit in a biomarker-defined population, indicating true dependency. In the SOLO1 phase III trial, patients who responded to platinum-based therapy and received olaparib maintenance experienced sustained long-term benefits over extended follow-up, suggesting that when HR deficiency is the primary lesion, PARP inhibitors can lead to long-lasting clinical benefit [159].

A second pattern of success is that combination maintenance can increase benefits when biology and selection align. In the PAOLA-1 phase III trial, adding olaparib to bevacizumab maintenance therapy provided substantial benefit for patients with HRD-positive disease, but no meaningful benefit was observed for patients with HRD-negative tumors [160]. This serves as an instructive reminder that combining agents does not compensate for a weak dependency if the tumor is not HRD-driven.

In prostate cancer, combination strategies demonstrate a more nuanced success profile. PARP inhibitor + ARPI combined regimens have demonstrated radiographic (rPFS) benefits in first-line mCRPC settings. In the PROpel phase III trial, olaparib plus abiraterone improved rPFS in an unselected mCRPC population, although interpretation of the magnitude and consistency of benefit across biomarker strata continues to evolve as overall survival data mature [161]. TALAPRO-2, a phase III trial, showed a significant rPFS benefit for talazoparib plus enzalutamide in both the molecularly unselected and HRR-deficient populations. Final overall survival analysis later demonstrated a significant OS benefit in the overall population, although the effect appeared more pronounced in HRR-deficient patients than in HRR-non-deficient/unknown patients [162]. Collectively, these trials support the translation of DDR concepts in mCRPC while simultaneously indicating that the most durable and clinically significant benefits tend to concentrate in tumors with clearer dependency signals.

#### 8.1.2 Failures

Where DDR translation has been mixed or has failed outright, the culprit is not usually an absence of target engagement. Rather, it is often dilution of dependency, biomarker ambiguity, and, most decisively, deliverability constraints that force biologically suboptimal exposure. This is particularly evident when eligibility criteria expand more quickly than dependency mapping.

The MAGNITUDE phase III trial tested niraparib plus abiraterone in metastatic castration-resistant prostate cancer (mCRPC) patients with prospective stratification by HRR status. The HRR-negative cohort was stopped for futility, and benefit was confined to HRR-altered tumors (most clearly BRCA1/2), illustrating how quickly efficacy collapses when PARP inhibitor-based combinations are pushed beyond dependency-defined populations [163]. More broadly, these programs demonstrate that combining treatments does not automatically compensate for weak dependency; instead, it can amplify toxicity.

Deliverability failures are even more pronounced in replication-stress kinase inhibition, where hematologic toxicity often limits continuous exposure. This raises a difficult question about how well the dosing schedules achieve the desired biological effects, or whether they merely make the treatments easier to tolerate without maximizing efficacy.

An additional and often underappreciated limitation in prostate cancer trials relates to tissue acquisition strategies used for biomarker stratification. In mCRPC, HRR status is frequently determined using archival primary tumor samples rather than contemporaneous metastatic biopsies. This approach introduces a fundamental source of misclassification, as the molecular landscape of advanced disease can diverge substantially from that of the primary tumor due to clonal evolution under therapeutic pressure [164,165]. In this context, reliance on historical tissue may fail to capture key resistance mechanisms, including BRCA reversion mutations or functional restoration of HR, leading to inaccurate assignment of HRR status at the time of treatment [165]. Moreover, spatial heterogeneity across metastatic sites further compounds this limitation, as different lesions may harbor distinct genomic and functional profiles. Consequently, biomarker-defined trial populations in mCRPC may be inherently diluted not only by broad eligibility criteria but also by structural limitations in tissue sampling, which weaken the biological precision of HRR-based stratification [165,166].

### 8.2 The Challenge of Tumor Heterogeneity and Clonal Evolution

The main challenge for DDR therapeutics is not a lack of possible resistance mechanisms, but the unavoidable presence of heterogeneity and continuous evolution under intense selection pressure. Under DDR-targeting regimens, tumors rarely follow a single linear path to escape. Instead, they often converge on a resistant phenotype through various routes that can evolve over time and across metastatic sites.

This is critically important because most biomarker strategies capture a dependency state at one time point and often from one anatomical compartment. In a disease that is dynamic and spatially heterogeneous, the true state is a moving target, and the translational risk is that a trial tests a biologically diluted population where only a minority of lesions remain dependent on the DDR node being targeted.

Mechanistically, the field has identified a wide range of resistance mechanisms to PARP inhibitors, including restoration of homologous recombination, stabilization of replication forks, and rewiring of DNA damage signaling [164,167]. These frameworks help explain why resistance is often multifactorial. BRCA reversion mutations are a clinically illustrative example of heterogeneity-driven resistance. These reversions are compelling because they directly restore gene function and reverse synthetic lethal dependence. However, their translational significance extends beyond the mechanism itself.

In a case report of mCRPC, Walmsley and colleagues documented convergent evolution of BRCA2 reversion mutations under PARP inhibitor pressure, illustrating extensive spatial and temporal heterogeneity across sites and time points [165]. These observations highlight two important points for translational design: resistance can be polyclonal and anatomically compartmentalized. Consequently, a biopsy of a single lesion may be informative yet incomplete. Even plasma-based assays can underrepresent resistant subclones when shedding is low or compartmentalization is pronounced.

### 8.3 Industrial Perspectives: Next-Generation DDR Inhibitors with Improved Properties

From an industry perspective, the goal is to transform DDR biology into scalable therapies. These therapies should consist of agents that can be administered at biologically meaningful exposure levels in real-world patients, combined rationally, and supported by biomarkers that remain feasible under multicenter variability. In that sense, next-generation DDR inhibitor development is less about discovering new targets and more about optimizing DDR therapies through improved selectivity to create dosing flexibility and optimized pharmacokinetics and pharmacodynamics.

PARP1-selective inhibitors are being developed to preserve antitumor potency while reducing toxicity. In the Saruparib translational study, the value proposition is not only that the agent is active in HRD models but also that its selectivity and durable activity support mechanistically coherent combinations, including with ATR inhibitors [166].

ATR inhibitors illustrate how industrial translation often hinges on development architecture as much as medicinal chemistry. Clinical synthesis has highlighted the heterogeneity of efficacy and the recurrence of hematologic toxicities, which limit feasible dosing, especially in combination therapies. This reinforces the importance of scheduling optimization and biomarker enrichment as prerequisites for achieving meaningful clinical impact [168,169].

## 9 Conclusion

DNA repair inhibitors, led by PARP inhibitors, have transformed treatment for homologous recombination-deficient tumors by establishing synthetic lethality as a clinically validated principle. However, resistance emerges through dynamic adaptive evolution encompassing genetic reversion, epigenetic reprogramming, replication fork stabilization, metabolic rewiring, and immune modulation. A central insight is that resistance creates new vulnerabilities. Tumors that restore homologous recombination often remain reliant on compensatory pathways such as POLQ-mediated end joining or exhibit elevated replication stress, rendering them susceptible to ATR, CHK1, or WEE1 inhibition. Epigenetic remodeling can be therapeutically reversed, and DNA damage-induced immunogenicity can be harnessed with immune checkpoint inhibitors. The therapeutic index of these combinations depends critically on schedule optimization and next-generation inhibitors. Clinically, managing resistance demands parallel evolution in biomarker strategies. Static, single-timepoint genomic assessments are insufficient. An integrated approach combining longitudinal ctDNA monitoring, functional RAD51 foci assays for real-time HR competence, and emerging imaging modalities for target engagement offers a path toward adaptive, biomarker-driven treatment selection.

## 10 Future Perspective

Looking forward, the field must move beyond the binary paradigm of sensitivity versus resistance toward an evolution-aware framework that anticipates and intercepts adaptive escape. This will require not only mechanistically rational combination regimens but also clinical trial designs that incorporate dynamic biomarker surveillance and permit treatment adaptation based on emerging resistance phenotypes. The industrial development of next-generation DDR inhibitors with improved pharmacokinetic properties and reduced toxicity will be essential to enable the sustained target engagement required for durable disease control. By embracing the complexity of tumor evolution and integrating molecular insights with real-time monitoring, the next decade of DDR-targeted therapy holds the promise of transforming synthetic lethality from a transient vulnerability into an enduring therapeutic liability for resistant cancers.

## Data Availability

Not applicable.
